# TssW-PpkA-Fha axis controls the positioning and initiation of the type VI secretion system in *Acidovorax citrulli*

**DOI:** 10.1128/mbio.01879-25

**Published:** 2025-09-22

**Authors:** Tong-Tong Pei, Xing-Yu Wang, Zi-Yan Ye, Yi-Qiu Zhang, Jing-Tong Su, Han Luo, Ya-Jie Zhao, Hao-Yu Zheng, Zhu Si, Ying An, Xiaoye Liang, Tao Dong

**Affiliations:** 1 School of Life Sciences, Southern University of Science and Technology, Shenzhen, Guangdong, China; 2 State Key Laboratory of Microbial Metabolism, Joint International Research Laboratory of Metabolic & Developmental Sciences, School of Life Sciences and Biotechnology, Shanghai Jiao Tong University 200639, Shanghai, China; 3 Cryo-electron Microscopy Center, Southern University of Science and Technology 255310, Shenzhen, Guangdong, China; Hebrew University of Jerusalem Robert H Smith Faculty of Agriculture Food and Environment, Rehovot, Israel

**Keywords:** protein secretion, LLPS, PpkA, phosphorylation, *Acidovorax*

## Abstract

Understanding how bacteria spatially regulate their molecular weaponry is key to unraveling their virulence and survival strategies in competitive ecosystems. The type VI secretion system (T6SS) is a highly potent contractile nanomachine that promotes bacterial survival by targeting competitors and hosts. While T6SS functionality has been extensively linked to the diversity of secreted effectors, the role of accessory components in spatially regulating its activity remains less understood. Here, we uncover that two membrane components, PpkA and TssW, are crucial modulators of T6SS efficacy in the plant pathogen *Acidovorax citrulli*, which possesses broad-spectrum antibacterial and antifungal activity. PpkA, an inner-membrane kinase, is essential for constitutive T6SS activity through its interactions with structural components and the assembly scaffold Fha. TssW, an outer-membrane anchor, determines T6SS positioning via interactions spanning the inner and outer membranes. Notably, PpkA promotes T6SS assembly by phosphorylating Fha and forming liquid-liquid phase-separated condensates that selectively recruit T6SS structural components at the membrane interface. These findings reveal a novel mechanism for accessory proteins in T6SS positioning and efficacy, highlighting the importance of spatial regulation in bacterial competition and virulence.

**IMPORTANCE:**

How cells determine where to assemble a macromolecular complex is a fundamental question in biology since the localization of these complexes is directly linked to functions. In bacteria, the type VI secretion system (T6SS) relies on effective positioning to target competitor and host cells in contact-dependent interactions. This study identifies a PpkA-TssW-Fha axis that orchestrates T6SS localization and activation through membrane anchoring and liquid-liquid phase separation at the inner membrane interface. These new insights can help us not only better understand how the T6SS functions but also better design T6SS-based solutions for therapeutic targeting of drug-resistant and T6SS-susceptible bacterial and fungal pathogens.

## INTRODUCTION

Microbes have developed a plethora of intricate macromolecular machinery that performs diverse functions, including cell division, motility, virulence, and host-microbe interactions (1–5). To fully understand how these protein machines operate, it is critical to not only identify their components but also determine their localization. Especially in the context of cell-cell interactions, the positioning of protein machines that mediate contact-dependent interactions is foreseeably critical to their functions. One such form of interaction is mediated by the type VI secretion system (T6SS), a molecular contractile spear that can be ejected by a donor cell to penetrate the cell envelopes of diverse cell types, including both gram-negative and gram-positive bacterial and fungal species (6–11).

The T6SS spear-like structure is anchored by a trans-envelope complex consisting of an outer-membrane protein (TssJ) and two inner-membrane proteins (TssL and TssM) (12, 13). The membrane complex (MC) connects to a TssE/F/G/K baseplate, a PAAR/VgrG spike, and a tubular structure composed of a TssB/C outer sheath and an Hcp inner tube (10, 12, 14–16). Some T6SSs contain less conserved yet crucial components, including ImpA_N family chaperones and a forkhead-associated domain protein (Fha) for assembly, and an AAA+ ATPase (ClpV) for disassembly (17–19). The T6SS spear delivers a wide variety of toxic effectors in different species, targeting both eukaryotic and prokaryotic species (5, 7, 20–24).

In addition to effector functions, the efficacy of the T6SS is closely linked to the spatial targeting of recipient cells (5, 25–27). The current model includes the constitutive firing of the T6SS into recipient cells and retaliatory attacks upon sensing a contact-dependent signal that may result from an incoming T6SS and conjugation, or other membrane-perturbing diffusible signals (27–29). While the constitutive firing is somewhat random, the latter resembles a unique precise response mediated by a trans-envelope regulatory module. In *Pseudomonas aeruginosa*, an important human pathogen, this module comprises TagQ (an outer-membrane lipoprotein), TagR (an outer membrane-associated protein), TagS and TagT (an inner-membrane complex), and the threonine kinase (PpkA)-phosphatase (PppA) signaling pathway (5, 18, 27). The TagQRST pathway activates the inner-membrane kinase PpkA, which subsequently phosphorylates Fha1 to trigger H1-T6SS assembly. Dephosphorylating Fha1 by PppA inactivates the T6SS. In species lacking the TagQRST module, PpkA constitutively activates the T6SS by phosphorylating Fha or TssL (30–32). However, our understanding of how PpkA mediates T6SS functions remains limited.

The T6SS is found in approximately 25% of gram-negative bacteria, exhibiting diverse functionalities (33). Among these, *Acidovorax citrulli* AAC00-1, a plant pathogen commonly found in cucurbit plants, encodes a single but highly potent T6SS capable of efficiently targeting a broad range of competitors, including gram-negative and gram-positive bacteria, as well as fungi (11). Its ability to penetrate the relatively thick cell envelope of *Bacillus subtilis* and fungal cells suggests unique features beyond a large repertoire of effectors (11). Our study aimed to elucidate the mechanisms underlying such efficiency by focusing on its T6SS assembly process. We demonstrate that an outer-membrane protein, TssW, is crucial for the localization and dynamics of the T6SS, through interacting with two inner membrane (IM) components, TssM and PpkA. PpkA functions as an inner membrane kinase that phosphorylates Fha and forms liquid-liquid phase separation (LLPS) condensates, which could selectively recruit T6SS components. TssW and PpkA thus serve as accessory structural components to the conserved T6SS architecture. These results collectively highlight an additional layer of regulation, involving localization control and LLPS condensate formation, for enhancing the T6SS efficacy and promoting bacterial competitive fitness in complex environments.

## RESULTS

### TssW is critical to T6SS activation and localization

**Fig 1:**
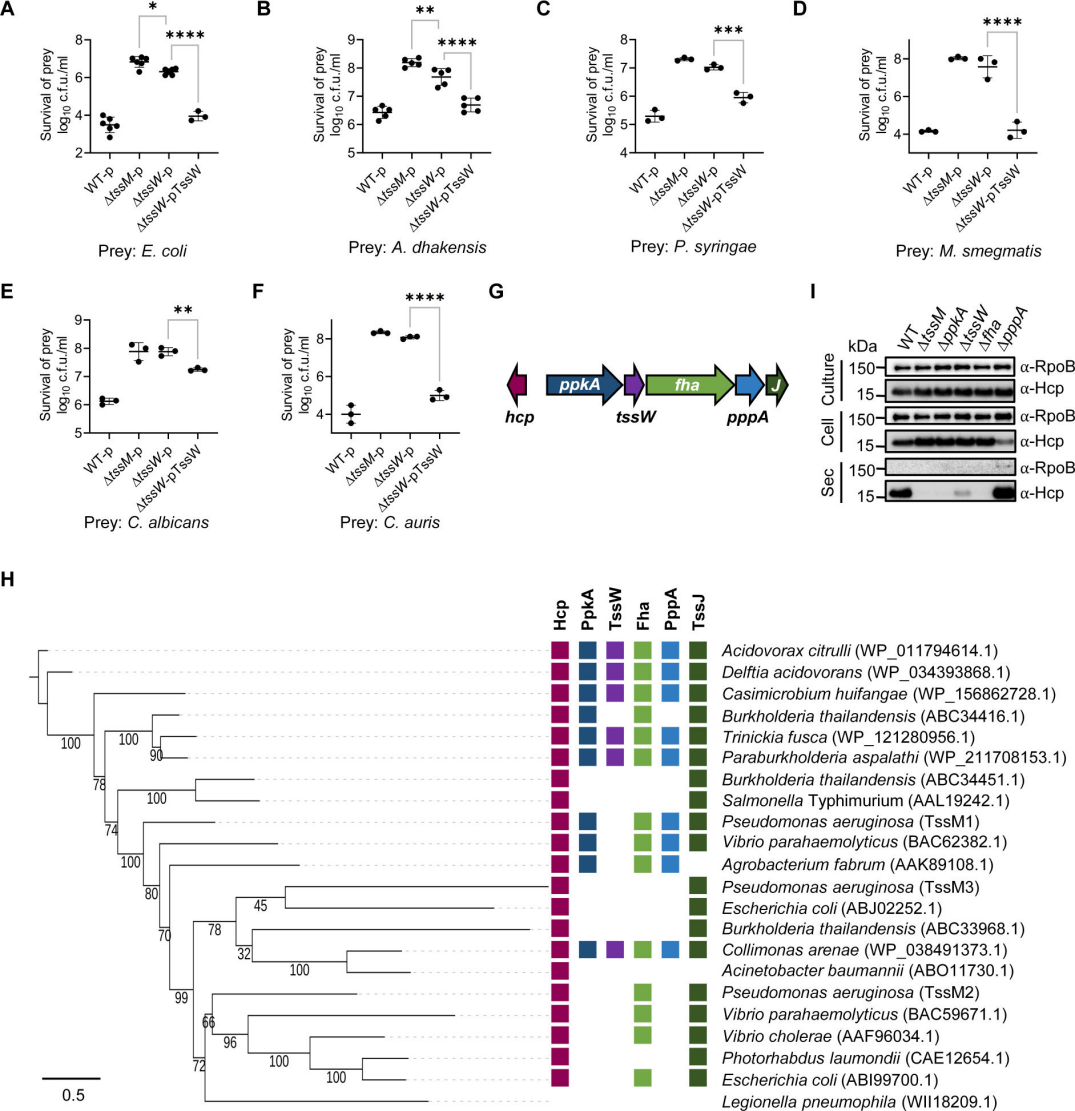
TssW plays a crucial role in the T6SS activity. Competition analysis of the AAC00-1 wild type (WT), T6SS-null mutant Δ*tssM*, Δ*tssW*, and Δ*tssW* mutant complemented with TssW against *E. coli* MG1655 (**A**), *A. dhakensis* SSU (**B**), *P. syringae* pv. *syringae* (**C**), *M. smegmatis* (**D**), *C. albicans* (**E**), and *C. auris* (**F**). Survival of killer strains during competition assays is depicted in Fig. S1A through F. For (**A**)**–**(**F**), error bars indicate the mean ± standard deviation of at least three biological replicates and statistical significance was calculated using one-way analysis of variance test for each group, *P < 0.05, **P < 0.01, ***P < 0.001, ****P < 0.0001. (**G**) Schematic depiction of *tssW* localization within the T6SS gene cluster in *A. citrulli* AAC00-1. (**H**) Relationship between phylogeny and T6SS gene content. On the left, the maximum likelihood phylogeny of TssM homologs is presented, constructed from aligned sequences using a bootstrap method with 1,000 replicates. Protein sequences are provided in Data S1. On the right, the presence of T6SS components Hcp, TssJ, PpkA, TssW, Fha, and PppA is depicted in columns, utilizing the same color code as presented in Fig. 1G. (**I**) Secretion analysis of Hcp in AAC00-1 WT, T6SS-null mutant Δ*tssM*, Δ*ppkA*, Δ*tssW*, Δ*fha*, and Δ*pppA*. RpoB serves as a control for equal loading and autolysis. The quantification of secreted Hcp proteins is shown in Fig. S1G.

Through genetic mutagenesis and competition assays, we identified a gene of unknown function, *tssW*, that is critical for the T6SS-mediated killing activities against *Escherichia coli*, *Aeromonas dhakensis*, *Pseudomonas syringae*, and *Mycobacterium smegmatis*, and two critical fungal pathogens *Candida albicans* and *Candida auris* (Fig. 1A through F; Fig. S1A through F). Deletion of *tssW* significantly impaired, but did not abolish, the T6SS-mediated killing ability, which was restored by plasmid-borne *tssW* (Fig. 1A through F; Fig. S1A through F). Genomic analysis shows that *tssW* is encoded within the T6SS cluster between *ppkA* and *fha*, which are further flanked by two conserved T6SS structural genes, *hcp* and *tssJ* (Fig. 1G). Comparative genomic analysis of this region reveals that *tssW* is associated with *ppkA* and *fha* in a number of T6SS species, including *Delftia acidovorans*, *Casimicrobium huifangae*, *Trinickia fusca*, *Paraburkholderia aspalathi*, and *Collimonas arenae* (Fig. 1H).

To understand the role of TssW in controlling T6SS functions, we performed protein secretion assays. Results show that deletion of *tssW* resulted in a substantial reduction in secreted Hcp levels, while cellular Hcp levels remained unchanged (Fig. 1I; Fig. S1G). Additionally, deletion of its neighboring genes *ppkA* and *fha* abolished Hcp secretion, while deletion of *pppA* likely increased Hcp secretion (Fig. 1I; Fig. S1G), which is consistent with previous observations of PpkA-PppA homologs in *P. aeruginosa* (18).

**Fig 2:**
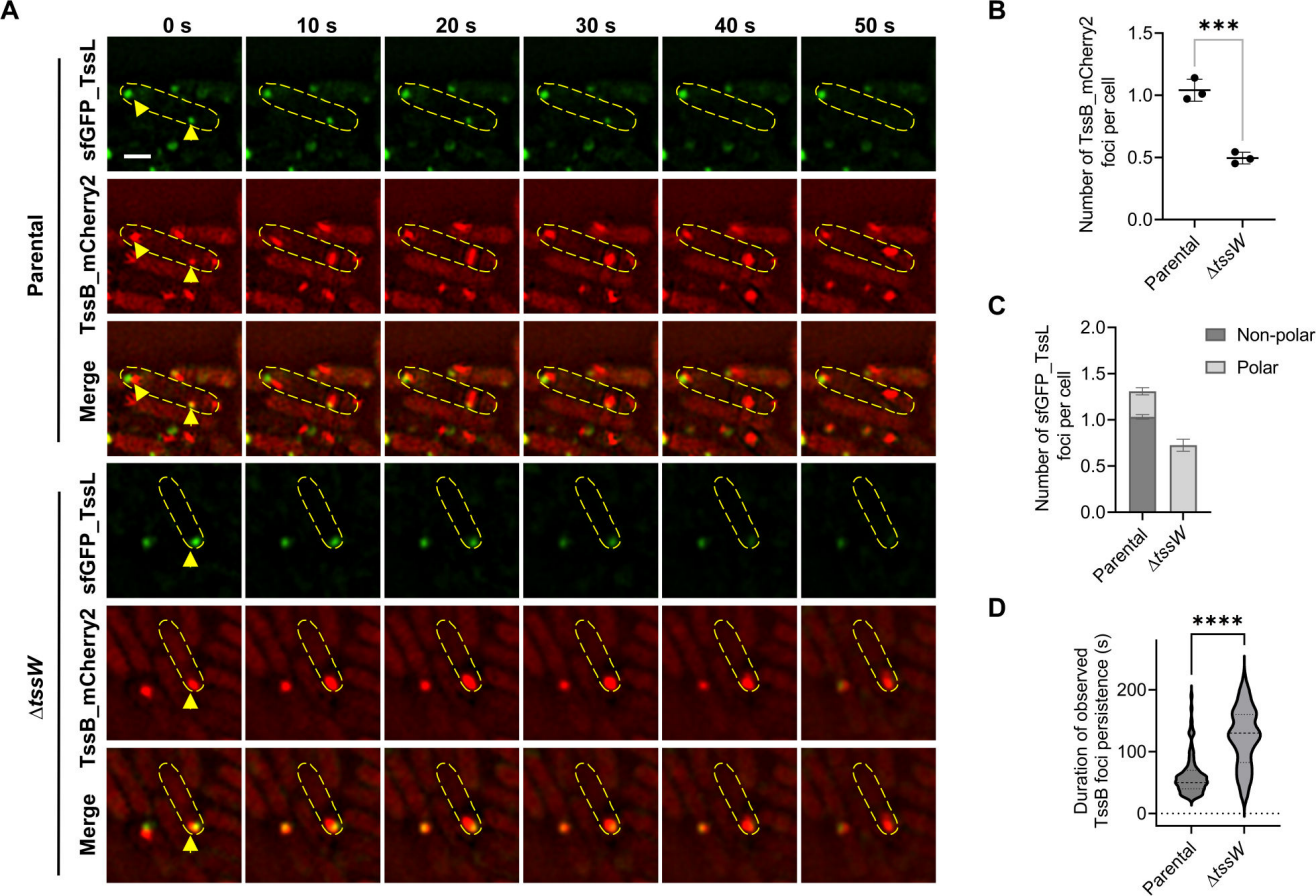
T6SS apparatuses become predominantly localized to the poles upon *tssW* deletion. (**A**) Time-lapse images showing colocalization between chromosomally encoded sfGFP_TssL and TssB_mCherry2 in AAC00-1 Parental and Δ*tssW*. Yellow arrows highlight representative localization of sfGFP_TssL and TssB_mCherry2. Yellow dashed lines depict the contour of the T6SS cell. Images were acquired using SIM. A representative 5 × 5 µm field of cells is shown. Scale bar: 1 µm. (**B**) Quantification of cells forming TssB_mCherry2 foci in AAC00-1 Parental and Δ*tssW*. Each data point represents the number of foci per cell quantified from an individual 33 × 33 µm field of view. (**C**) Quantification of cells forming sfGFP_TssL foci in AAC00-1 Parental and Δ*tssW*. The ratios of polar and non-polar foci are shown in light gray and dark gray, respectively. (**D**) Quantification of the persistence duration of TssB foci in AAC00-1 Parental and Δ*tssW*. A total of 100 TssB foci were analyzed for both parental and Δ*tssW*. For (**B**)**–**(**D**), error bars indicate the mean ± standard deviation of at least three biological replicates, and statistical significance was calculated using a two-tailed Student’s *t*-test. ***P < 0.001, ****P < 0.0001.

We then examined the effect of *tssW* deletion on T6SS dynamics by time-lapse imaging of mCherry2-labeled TssB (sheath) and sfGFP-labeled TssL (membrane complex) using both structured illumination microscopy (SIM) and wide-field fluorescence microscopy. Deletion of *tssW* decreased the number of TssB and TssL foci per cell and restricted the TssL foci to the cell poles (Fig. 2A through C; Fig. S2A; Movie S1). Moreover, individual TssB foci in the Δ*tssW* mutant persisted for longer durations (Fig. 2A and D; Movie S1), indicating a slower assembly-contraction cycle. Deletion of *tssW* also resulted in polar localized Fha_sfGFP foci (Fig. S2B). These results collectively suggest that TssW plays a crucial role in T6SS activation and localization.

### TssW is an outer-membrane protein interacting with the T6SS inner-membrane complex

**Fig 3:**
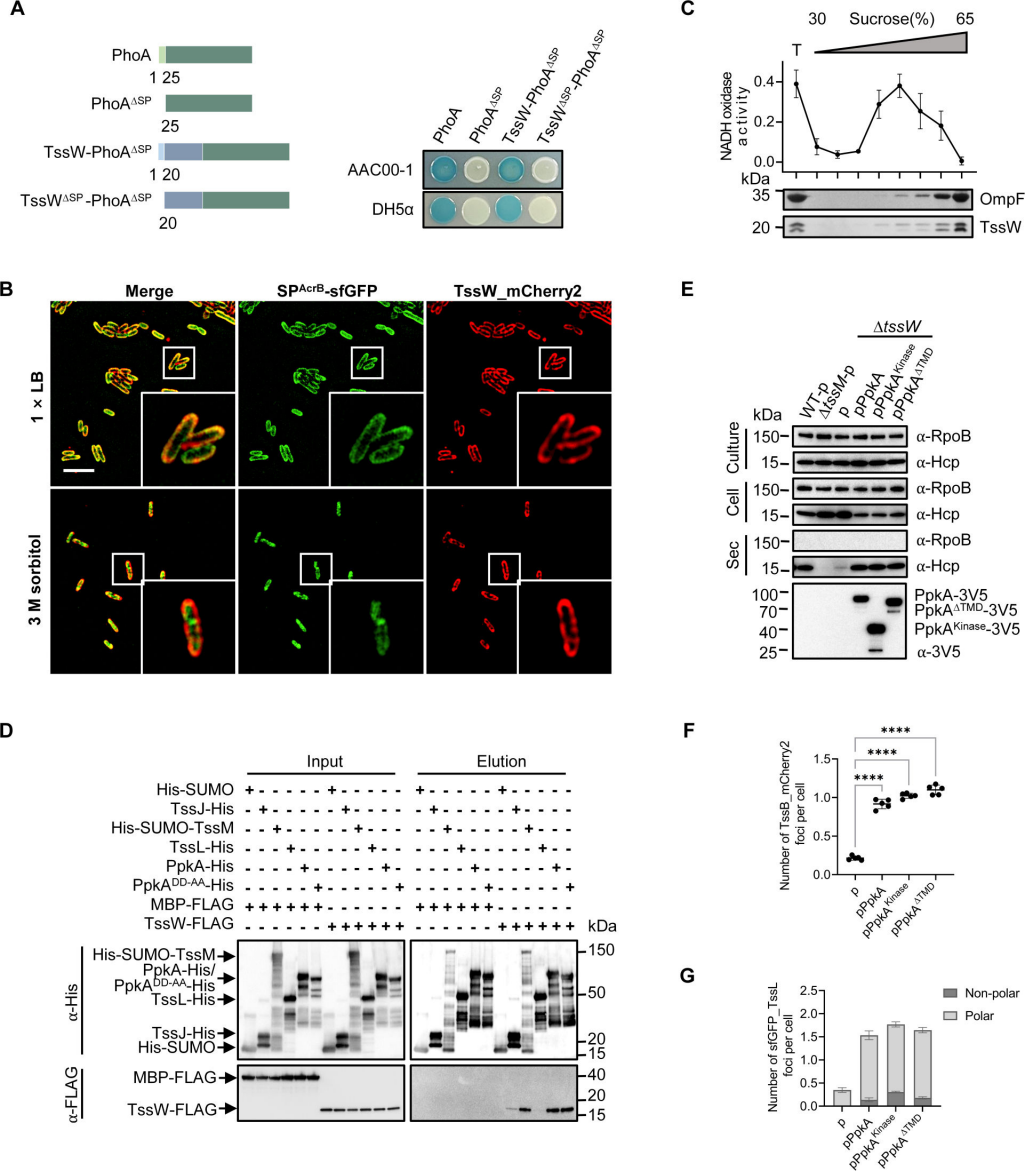
Outer membrane-spanning TssW facilitates non-polar T6SS localization. (**A**) Alkaline phosphatase activity analysis of PhoA, PhoA^ΔSP^, TssW-PhoA^ΔSP^, and TssW^ΔSP^-PhoA^ΔSP^ in *A. citrulli* AAC00-1 and *E. coli* DH5α. The PhoA fusion assay was used to assess the periplasmic localization of the constructs, indicated by the blue color resulting from BCIP cleavage in the periplasm. The diagram illustrates the used constructs (left). PhoA^ΔSP^ and TssW^ΔSP^ deleted their natural Sec signal, respectively. Cells carrying plasmid-borne PhoA, PhoA^ΔSP^, TssW-PhoA^ΔSP^, or TssW^ΔSP^-PhoA^ΔSP^ were grown on lysogeny broth (LB) agar plates with BCIP. For *E. coli* DH5α strains, 0.01% L-arabinose was also included to induce expression from the pBAD vector. PhoA and PhoA^ΔSP^ serve as positive and negative controls, respectively, with PhoA localized to the periplasm and PhoA^ΔSP^ unable to reach the periplasm due to the lack of its Sec signal peptide. (**B**) SIM images showing the TssW_mCherry2 and SP^AcrB^-sfGFP localization in AAC00-1. Live cells were imaged after treatment with 1 × LB or plasmolysis in 3 M sorbitol. A representative 30 × 30 µm field of cells with a 3× magnified 5 × 5 µm inset (marked by box) is shown. Scale bar: 5 µm. (**C**) TssW cofractionates with the outer membrane. Total (**T**) membranes from AAC00-1 cells expressing TssW-3V5 were separated using a discontinuous sucrose sedimentation gradient. The collected fractions were analyzed by Western blotting with ⍺-OmpF and ⍺-V5 antibodies (from top to bottom panel). OmpF was used as a control for the outer membrane, while NADH oxidase activity served as a control for the inner membrane (upper graph). (**D**) Interaction of TssW with TssJ, TssM, PpkA, and PpkA^DD-AA^. Pull-down analysis was performed using His-tagged SUMO (control), TssJ, SUMO-TssM, TssL, PpkA or PpkA^DD-AA^, and FLAG-tagged maltose binding protein (MBP) (control) or TssW. (**E**) Secretion analysis of Hcp in AAC00-1 Δ*tssW* expressing PpkA, PpkA^ΔTMD^, or PpkA^Kinase^. RpoB serves as a control for equal loading and autolysis. The quantification of secreted Hcp proteins is shown in Fig. S3A. (**F**) Quantification of cells forming TssB_mCherry2 foci in AAC00-1 Δ*tssW* expressing PpkA, PpkA^Kinase^, or PpkA^ΔTMD^. Each data point represents the number of foci per cell quantified from an individual 33 × 33 µm field of view. (**G**) Quantification of cells forming sfGFP_TssL foci in AAC00-1 Δ*tssW* expressing PpkA, PpkA^Kinase^, or PpkA^ΔTMD^. The ratios of polar and non-polar foci are shown in light gray and dark gray, respectively. For (**F**)** and **(**G**), error bars indicate the mean ± standard deviation of five biological replicates, and statistical significance was calculated using one-way analysis of variance test for each group, ****P < 0.0001.

To elucidate how TssW functions, we first determined its subcellular localization, noting the presence of an N-terminal secretion signal. We employed the alkaline phosphatase (PhoA) reporter system. When PhoA is directed to the periplasm, either by its native secretion signal or a carrier protein of interest, it cleaves the inner membrane-impermeable substrate 5-bromo-4-chloro-3-indolyl phosphate (BCIP) to produce a blue color, whereas cytoplasmic localization of PhoA results in no detectable activity (34, 35). Expression of PhoA in both *A. citrulli* AAC00-1 and *E. coli* DH5α led to periplasmic degradation of the substrate BCIP, whereas its PhoA^ΔSP^ mutant, lacking the native secretion peptide, did not degrade BCIP (Fig. 3A). When TssW was fused to the N-terminus, but not the C-terminus, of PhoA^ΔSP^, BCIP-degrading activity was detected. This suggests the C-terminus of TssW is localized in the periplasm (Fig. 3A).

To determine the localization of TssW, we constructed a chromosomal fusion of TssW_mCherry2 in *A. citrulli*. As an inner-membrane protein control, we also introduced a plasmid-borne sfGFP fused with the N-terminal signal peptide of AcrB (Aave_2528), a conserved inner-membrane protein, into the *tssW*_*mCherry2* strain. Imaging results showed that TssW_mCherry2 and SP^AcrB^-sfGFP were distributed on the cell membranes (Fig. 3B). After treatment with 3 M sorbitol to induce hyperosmotic stress and cytoplasmic shrinkage (36), the distribution of TssW_mCherry2 remained unchanged while the signals of SP^AcrB^-sfGFP were condensed, suggesting that TssW is localized on the outer membrane (Fig. 3B). As a control, we tested the T6SS functions of the *tssW*_*mCherry2* strain in a competition assay. Although less than the wild type, it exhibited significantly greater T6SS-mediated killing activity than the Δ*tssW* mutant, indicating that this fusion is functional (Fig. S2C and D). To further validate the outer membrane localization of TssW, we employed sedimentation density gradient centrifugation to separate membrane fractions from *A. citrulli* AAC00-1. The presence of TssW in the outer membrane was confirmed by comparing its distribution to that of OmpF, a well-established outer membrane marker (Fig. 3C). The inner membrane fractions were verified using the NADH oxidase enzymatic assay, confirming the successful fractionation of membrane components (Fig. 3C) (37).

Next, we examined the interaction of TssW with known T6SS membrane proteins. Pull-down assays showed that TssW strongly interacts with TssM, PpkA, and its predicted catalytically inactive mutant at the conserved catalytic residues D161 and D179 (PpkA^DD-AA^) (Fig. 3D). However, the interaction between TssW and the outer membrane component TssJ was relatively weak, and no interaction was detected with TssL (Fig. 3D). Bacterial two-hybrid assays further confirmed interactions between TssW and the periplasmic domains of TssJ, TssM, and PpkA (Fig. S2E). We also detected an interaction between TssW and the periplasmic domain of TssL by bacterial two-hybrid assays (Fig. S2E), suggesting that the TssW-TssL interaction may be relatively weak or transient.

We hypothesize that the T6SS defect of *tssW* deletion might be due to inefficient PpkA activation, which might be compensated by overexpression of PpkA. Indeed, expressing full-length PpkA, its kinase domain only PpkA^Kinase^, or its transmembrane-domain deletion PpkA^ΔTMD^ mutant in the Δ*tssW* mutant restored the secretion of Hcp to the wild-type level (Fig. 3E; Fig. S3A). The assembly of TssB_mCherry2 sheath was also significantly increased (Fig. 3F; Fig. S3B; Movie S2). Interestingly, despite the increased number of T6SS apparatuses per cell, the T6SS membrane complex foci were still primarily localized to cell poles (Fig. 3G; Fig. S3B; Movie S2). We further compared the localization of chromosomal fusion of sfGFP_PpkA in *A. citrulli* wild-type and the Δ*tssW* strains. In wild-type cells, sfGFP_PpkA formed multiple discrete foci that appeared along the central axis of the cell (Fig. S3C). In contrast, in the Δ*tssW* mutant, these foci exhibited polar localization (Fig. S3C). Notably, the kinase-inactive PpkA^DD-AA^ mutant also exhibited predominantly polar foci in the absence of TssW, suggesting that this spatial distribution is independent of PpkA catalytic activity (Fig. S3C). Expression of plasmid-borne TssW in the Δ*tssW* mutant restored the formation of PpkA foci with non-polar localization (Fig. S3D and E). Collectively, TssW is critical for the proper localization of PpkA and the T6SS membrane complex.

### PpkA acts upstream of Fha for T6SS assembly

**Fig 4:**
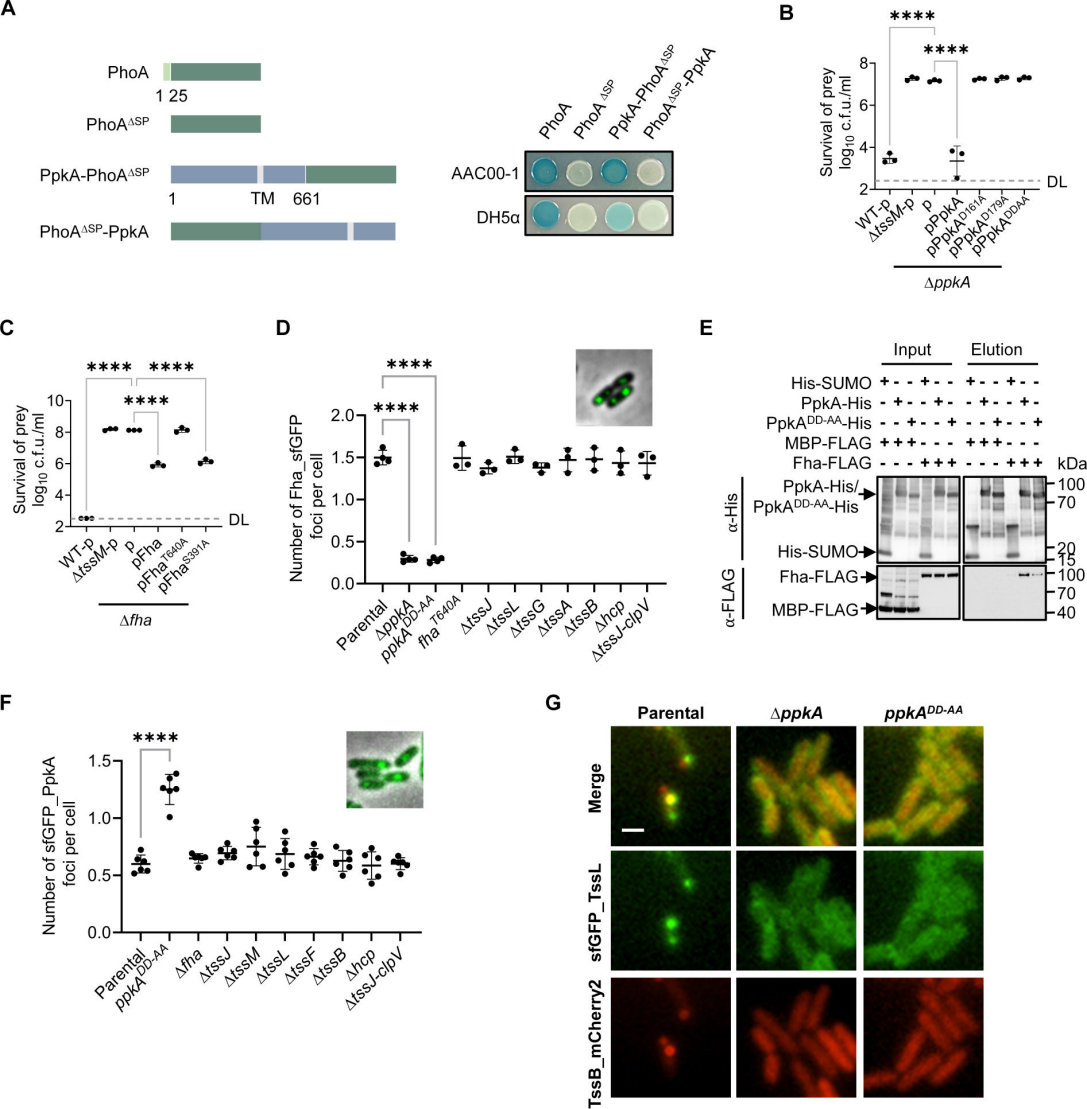
PpkA-mediated phosphorylation promotes Fha polymerization prior to T6SS assembly. (**A**) Alkaline phosphatase activity analysis of PhoA, PhoA^ΔSP^, PpkA-PhoA^ΔSP^, and PhoA^ΔSP^-PpkA in *A. citrulli* AAC00-1 and *E. coli* DH5α. The PhoA fusion assay was used to assess the periplasmic localization of the constructs, indicated by the blue color resulting from BCIP cleavage in the periplasm. The diagram illustrates the used constructs (left). PhoA^ΔSP^ deleted its natural Sec signal. *E. coli* DH5α cells carrying plasmid-borne PhoA, PhoA^ΔSP^, PpkA-PhoA^ΔSP^, and PhoA^ΔSP^-PpkA were grown on LB (lysogeny broth) agar plates with BCIP. For *E. coli* DH5α strains, 0.01% L-arabinose was also included to induce expression from the pBAD vector. PhoA and PhoA^ΔSP^ serve as positive and negative controls, respectively, with PhoA localized to the periplasm and PhoA^ΔSP^ unable to reach the periplasm due to the lack of its Sec signal peptide. (**B**) Competition analysis of the Δ*ppkA* mutant complemented with different PpkA mutants. Survival of killer strains during competition assays and expression of PpkA, PpkA^D161A^, PpkA^D179A^, and PpkA^DD-AA^ are depicted in Fig. S4A and B. (**C**) Competition analysis of the Δ*fha* mutant complemented with different Fha mutants. Survival of killer strains during competition assays and expression of Fha, Fha^T640A^, and Fha^S391A^ are depicted in Fig. S4C and D. For (**B) and (C**), killer strains are indicated, and the prey strain is the *E. coli* MG1655 carrying pPSV37-sfGFP plasmid. The WT and T6SS-null mutant Δ*tssM* serve as positive and negative controls, respectively. DL, detection limit. (**D**) Quantification of cells forming Fha_sfGFP foci in AAC00-1 Parental, Δ*ppkA*, *ppkA^DD-AA^*, *fha^T640A^*, Δ*tssJ*, Δ*tssL*, Δ*tssG*, Δ*tssA*, Δ*tssB*, Δ*hcp*, and Δ*tssJ-clpV*. The formation of Fha_sfGFP foci in the AAC00-1 Parental strain is shown in the top right corner. (**E**) Interaction of Fha with PpkA and PpkA^DD-AA^. Pull-down analysis was performed using His-tagged SUMO (control), PpkA, or PpkA^DD-AA^ and FLAG-tagged MBP (maltose binding protein, control) or Fha. (**F**) Quantification of cells forming sfGFP_PpkA foci in AAC00-1 Parental, *ppkA^DD-AA^*, Δ*fha*, Δ*tssJ*, Δ*tssM*, Δ*tssL*, Δ*tssF*, Δ*tssB*, Δ*hcp*, and Δ*tssJ-clpV*. The formation of sfGFP_PpkA foci in the AAC00-1 Parental strain is shown in the top right corner. For (**D**)** and **(**F**), each data point represents the number of foci per cell quantified from an individual 33 × 33 μm field of view. For (**B–D** and **F**), error bars indicate the mean ± standard deviation of at least three biological replicates, and statistical significance was calculated using one-way analysis of variance test for each group, ****P < 0.0001. (**G**) Fluorescence microscopy images showing sfGFP_TssL and TssB_mCherry2 localization in AAC00-1 Parental, Δ*ppkA*, and *ppkA^DD-AA^*. Genotypes are indicated at the top. Scale bar: 1 µm.

In *A. citrulli*, PpkA has an N-terminal kinase domain, a transmembrane domain, and a periplasmic domain, similar to its homolog in *P. aeruginosa*, despite low sequence identity (26.13%). Using the PhoA reporter, we confirmed PpkA’s C-terminal periplasmic presence by showing it directs PhoA to the periplasm as a C-terminal, but not N-terminal, fusion (Fig. 4A). Based on the known PpkA-Fha interaction and phosphorylation in other species, we constructed a non-phosphorylatable Fha mutant (T640A) in addition to kinase-inactivated PpkA^DD-AA^. To confirm the critical role of PpkA in T6SS functions, we introduced plasmid-borne PpkA and its mutants into the Δ*ppkA* strain and tested T6SS-mediated competition against *E. coli*. Only wild-type PpkA restored the killing activity (Fig. 4B; Fig. S4A and B). Similarly, we introduced plasmid-borne Fha and its mutants (T640A and S391A) into the Δ*fha* strain. Complementation was observed for wild-type Fha and the S391A mutant, which is a predicted non-conserved phosphorylation site (Fig. 4C; Fig. S4C and D). In contrast, the T640A mutant failed to restore T6SS function (Fig. 4C; Fig. S4C and D). These findings confirm that PpkA kinase activity and phosphorylation of Fha at T640 are required for T6SS functions.

We recently reported that Fha is essential for recruiting T6SS components and initiating assemblies (38). Therefore, we next tested the effect of PpkA on Fha localization. Using a chromosomal Fha_sfGFP fusion and fluorescence microscopy, we found that deletion of *ppkA* or its catalytic inactivation significantly reduced the number of Fha_sfGFP foci, leading to a tendency for Fha_sfGFP foci to form predominantly at the cell poles (Fig. 4D; Fig. S4E). However, the T640A mutation, which abolishes the phosphorylation of Fha, did not affect Fha foci formation (Fig. 4D). Additionally, deletion of T6SS structural genes (*tssJ*, *tssL*, *tssG*, *tssA*, *tssB*, and *hcp*) or the entire region from *tssJ* to *clpV* had no impact on the formation of Fha_sfGFP foci in comparison to the wild-type strain (Fig. 4D).

The observation that Fha foci formation is independent of Fha phosphorylation but requires enzymatically active PpkA is intriguing. We hypothesized that the interaction of PpkA with Fha is important for the foci formation and modulated by its kinase activity. Using pull-down analysis, we detected interaction between Fha and PpkA, and the kinase catalytic mutation significantly weakened such interaction (Fig. 4E). This result suggests that the kinase catalytic residues stabilize the interaction of PpkA and Fha.

To visualize the colocalization of PpkA with Fha and the T6SS apparatus, we constructed chromosomally tagged strains *sfGFP_ppkA fha_mCherry2* and *sfGFP_ppkA mCherry2_tssL*. Both displayed significant T6SS-mediated killing ability, albeit at a reduced level compared with the wild type (Fig. S4F and G). Fluorescence microscopy revealed that sfGFP_PpkA formed discrete foci that colocalized with both Fha_mCherry2 and mCherry2_TssL (Fig. S4H and I), confirming their spatial association within the cell. Additionally, sfGFP_PpkA also formed foci in a panel of T6SS gene deletion strains, including the Δ*fha* mutant and the Δ*tssJ-clpV* mutant lacking multiple structural genes (Fig. 4F). Interestingly, the PpkA^DD-AA^-sfGFP appeared to have an increased number of foci (Fig. 4F). Deletion and catalytic mutation of *ppkA* abolished the assembly of TssL foci and TssB foci in *A. citrulli* cells (Fig. 4G). Furthermore, while Δ*fha* mutants failed to form baseplate complex foci as previously reported (38), the absence of *ppkA* did not abolish the formation of sfGFP_TssG foci, which predominantly localized at the cell poles in the Δ*ppkA* mutant that mimics effects of the Δ*tssW* deletion (Fig. S4J). These findings, together with the role of Fha in T6SS initiation, suggest that PpkA activation acts upstream of Fha for initiating the T6SS assembly.

### PpkA recruits T6SS components and forms condensates

**Fig 5:**
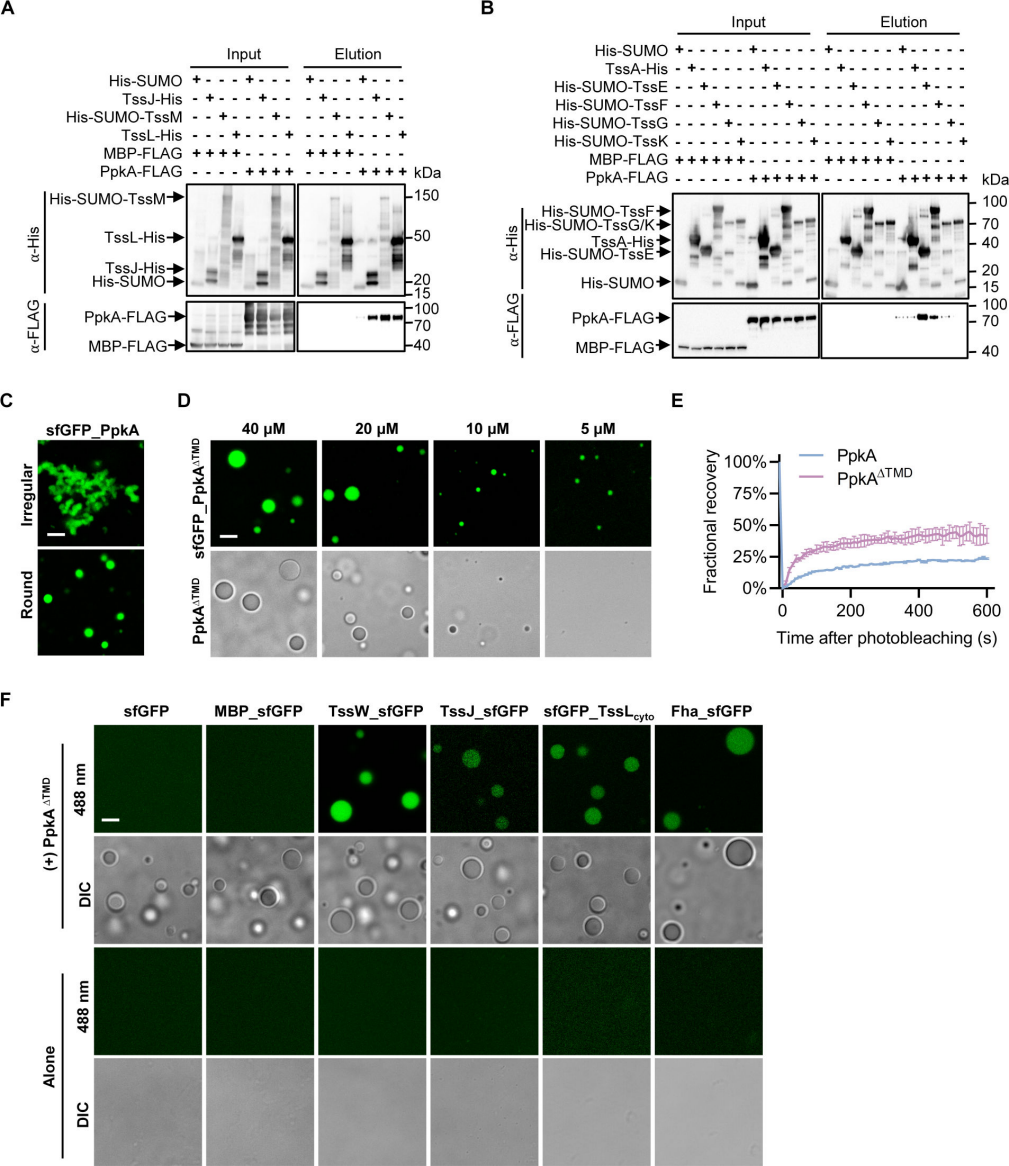
PpkA undergoes LLPS and selectively recruits T6SS components. (**A**) Interaction of PpkA with TssJ, TssM, and TssL. Pull-down analysis was performed using His-tagged SUMO (control), TssJ, SUMO-TssM, or TssL and FLAG-tagged MBP (maltose binding protein, control) or PpkA. (**B**) Interaction of PpkA with TssA, TssE, TssF, TssG, and TssK. Pull-down analysis was performed using His-tagged SUMO (control), TssA, SUMO-TssE, SUMO-TssF, SUMO-TssG, or SUMO-TssK, and FLAG-tagged MBP (control) or PpkA. (**C**) Fluorescence images showing LLPS of sfGFP_PpkA. A final concentration of 40 µM sfGFP_PpkA proteins in a 5% dextran-70 solution was employed. (**D**) Images showing LLPS of sfGFP_PpkA^ΔTMD^ and PpkA^ΔTMD^ at the indicated concentrations. (**E**) Quantification of the fluorescence recovery after photobleaching analyses for three different sfGFP_PpkA or sfGFP_PpkA^ΔTMD^ droplets. (**F**) Fluorescence images showing PpkA^ΔTMD^ recruited a number of T6SS-associated client proteins via LLPS *in vitro*. PpkA^ΔTMD^ proteins were mixed with sfGFP, MBP_sfGFP, TssW_sfGFP, TssJ_sfGFP, sfGFP_TssL_cyto_, and Fha_sfGFP at a ratio of 10:1 in a 10% dextran-70 solution before imaging. A representative 30 × 30 µm field is shown. Scale bar: 5 µm.

To further understand the role of PpkA, we tested whether PpkA interacts with other T6SS structural components using pull-down analysis. Of the His-tagged membrane complex components TssJ/M/L, PpkA exhibited clear interaction with all three of them (Fig. 5A). Of the baseplate components, PpkA exhibited strong interaction with TssE and TssF (Fig. 5B). These results suggest that PpkA forms a complex with the T6SS membrane and baseplate complex.

We recently demonstrated that Fha forms LLPS condensates that recruit T6SS client proteins (38). Since PpkA can also form foci and promote Fha foci formation in AAC00-1, we hypothesized that PpkA itself undergoes LLPS. To test this, we purified N-terminal sfGFP-tagged PpkA (sfGFP_PpkA) and its mutants lacking the transmembrane domain (sfGFP_PpkA^∆TMD^), the latter exhibiting increased solubility. We observed the formation of PpkA condensates in mixed states, including irregular shapes and round droplets in a dextran-70 supplemented buffer (Fig. 5C), a volume-excluding polymer used to mimic intracellular environments (39). The sfGFP_PpkA^∆TMD^ mutant exhibited better solubility, faster fluorescence recovery after photobleaching (FRAP), and formed droplets to about 5 µM concentrations (Fig. 5D and E; Fig. S4K). Untagged PpkA^∆TMD^ also exhibited droplet formation (Fig. 5D).

Next, we tested whether the PpkA^∆TMD^ droplet could recruit other T6SS components. Using purified sfGFP-labeled TssW (TssW_sfGFP), TssJ (TssJ_sfGFP), the cytoplasmic domain of TssL (sfGFP_TssL_cyto_), and Fha (Fha_sfGFP), we found that TssW_sfGFP, TssJ_sfGFP, sfGFP_TssL_cyto_, and Fha_sfGFP all colocalized in the PpkA^∆TMD^ droplets (Fig. 5F). In contrast, neither sfGFP nor sfGFP-labeled maltose binding protein (MBP_sfGFP) exhibited any colocalization (Fig. 5F). This suggests that PpkA droplets can selectively recruit these T6SS proteins independently of other proteins.

These results demonstrate that PpkA undergoes phase separation and selectively recruits T6SS components.

### TssW-PpkA-Fha axis modulates the localization and activities of the T6SS assembly

**Fig 6:**
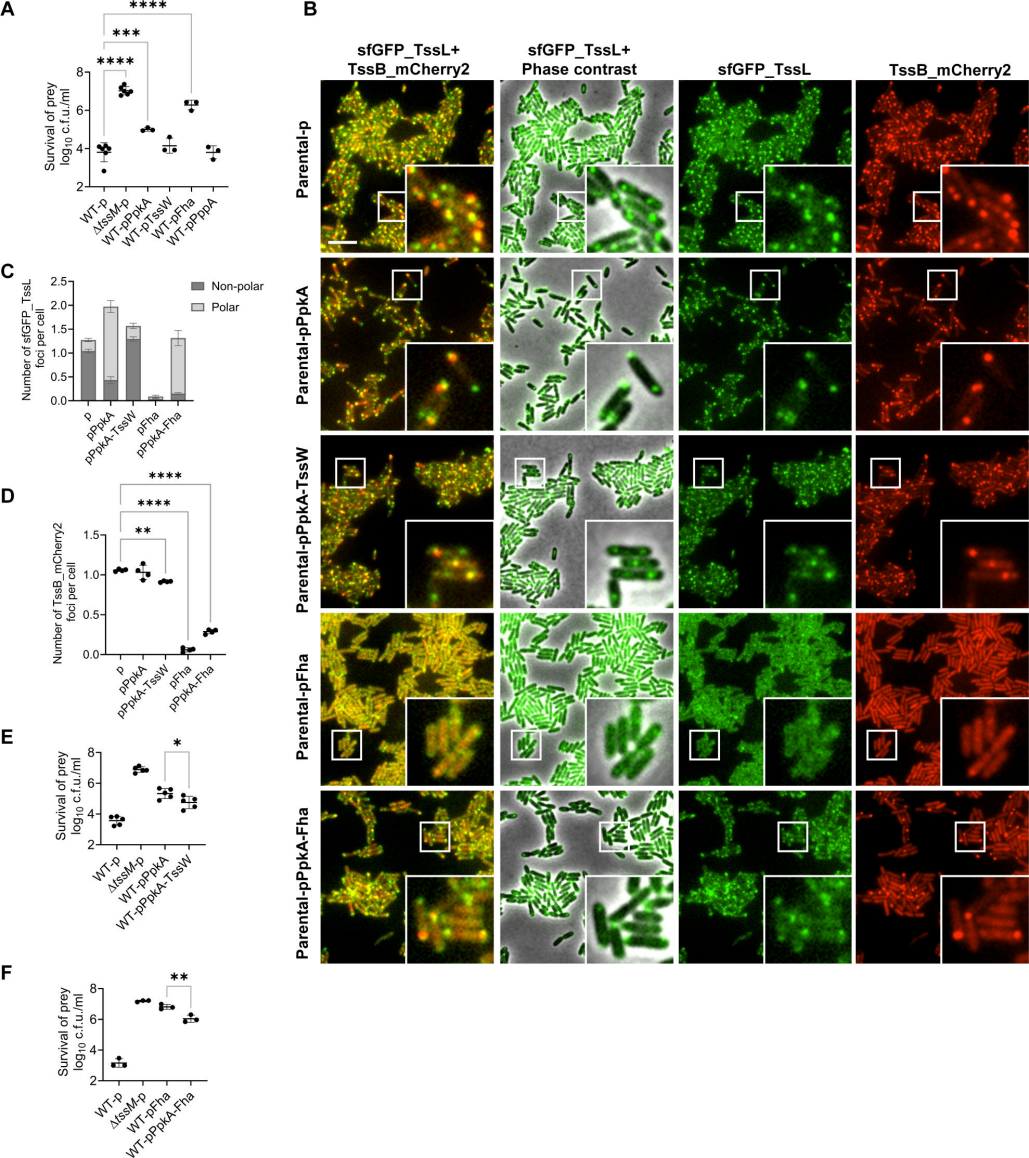
The stoichiometry of TssW, PpkA, and Fha regulates T6SS in AAC00-1. (**A, E,** and **F**) Competition analysis of the AAC00-1 wild type (WT) expressing different T6SS components. Survival of killer strains during competition assays and expression of PpkA, TssW, Fha, and PppA are depicted in Fig. S5. (**B**) Fluorescence microscopy images showing sfGFP_TssL and TssB_mCherry2 localization in AAC00-1 Parental strains expressing PpkA, TssW, or Fha. A representative 33 × 33 µm field of cells with a 3× magnified 5.5 × 5.5 µm inset (marked by box) is shown. Scale bar: 5 µm. (**C**) Quantification of cells forming sfGFP_TssL foci in AAC00-1 Parental strains expressing PpkA, TssW, or Fha. The ratios of polar and non-polar foci are shown in light gray and dark gray, respectively. (**D**) Quantification of cells forming TssB_mCherry2 foci in AAC00-1 Parental strains expressing PpkA, TssW, or Fha. Each data point represents the number of foci per cell quantified from an individual 33 × 33 μm field of view. For (**A**), (**C**)–(**E**), and (**F**), error bars indicate the mean ± standard deviation of at least three biological replicates, and statistical significance was calculated using one-way analysis of variance test for each group, *P < 0.05, **P < 0.01, ***P < 0.001, ****P < 0.0001.

To further explore how PpkA and TssW modulate T6SS assembly, we expressed plasmid-borne *ppkA, tssW,* and *fha* in wild-type *A. citrulli*. Bacterial competition assays showed that, while expressing TssW and PppA had little effect on the T6SS-mediated killing, the T6SS killing was reduced by PpkA induction and severely impaired by Fha induction (Fig. 6A; Fig. S5A and B). To investigate how PpkA and Fha expression affected T6SS killing, we monitored the assembly of the membrane complex and sheath by introducing these PpkA-TssW-Fha plasmids in *A. citrulli* carrying chromosomal sfGFP-TssL and TssB-mCherry2 fusions. Fluorescence microscopy analyses showed that expressing Fha severely reduced TssL foci formation, which was restored by co-expression of PpkA (Fig. 6B and C; Movies S3 and S4). Expression of PpkA alone stimulated TssL foci formation but resulted in biased polar localization, whereas co-expression of PpkA and TssW restored wild type-like distribution and increased T6SS-mediated killing abilities compared with expressing PpkA alone (Fig. 6B through E; Fig. S5C and D; Movies S3, S5 and S6). Additionally, the number of sheath formations was minimally affected by the expression of PpkA and TssW (Fig. 6B and D; Movies S3, S5 and S6). Expression of Fha substantially inhibited sheath formation, while co-expression of PpkA and Fha promoted the sheath assembly and T6SS-mediated killing ability relative to expressing Fha alone (Fig. 6B, D and F; Fig. S5D and E; Movies S4 and S7). To assess the expression levels of PpkA, TssW, and Fha from plasmid constructs, qRT-PCR was performed to measure the transcript levels in the corresponding *A. citrulli* strains. Transcription of each gene was specifically elevated upon plasmid-mediated expression (Fig. S5F through H). Western blotting analysis confirmed that Fha protein levels were elevated upon plasmid-based expression, with comparable levels observed between strains expressing Fha alone or co-expressing PpkA-Fha (Fig. S5I). These results are consistent with the model that Fha phosphorylation by PpkA is important for T6SS functions and highlight the critical role of TssW in regulating T6SS localization and functions via PpkA and Fha.

## DISCUSSION

**Fig 7:**
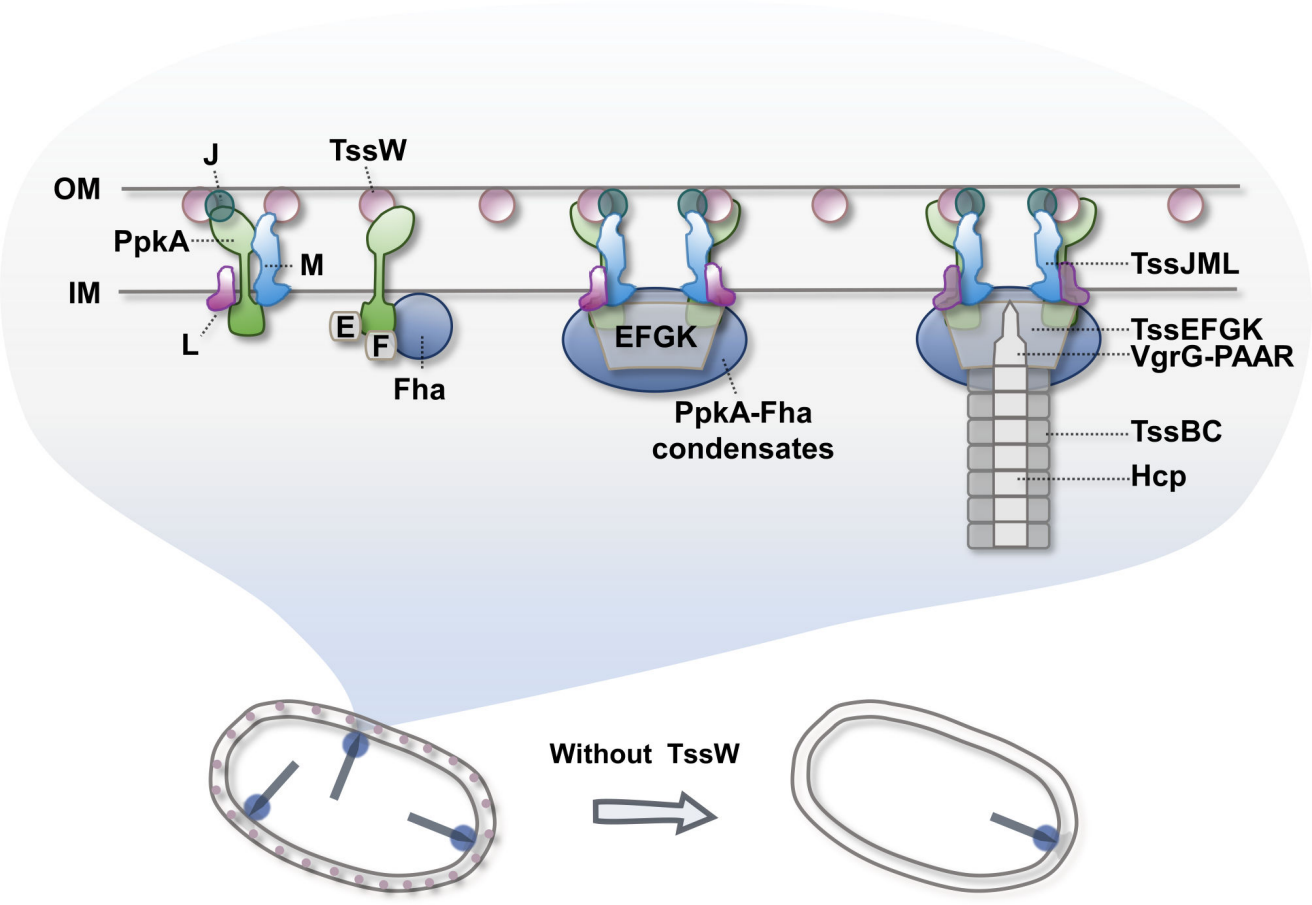
Schematic model for the role of TssW-PpkA-Fha in controlling T6SS assembly. In *A. citrulli*, TssW is distributed evenly on the OM and directly interacts with OM protein TssJ, IM protein TssM, and the kinase PpkA. PpkA interacts with TssJ, TssM, TssL, and phosphorylates Fha, which is essential for the assembly of the T6SS membrane complex. Both PpkA and Fha form LLPS condensates, selectively recruiting T6SS-associated components. Without TssW, T6SS structures in *A. citrulli* AAC00-1 are predominantly localized to the cell poles, underscoring the essential role of TssW as an OM anchor in promoting the formation of non-polar T6SS structures.

T6SS is a widespread molecular weapon commonly associated with human and plant pathogens and commensal gut microbes (33, 40, 41). In this study, we report an accessory axis that governs the localization and assembly of the T6SS in the plant pathogen *A. citrulli* (Fig. 7). TssW, a newly characterized T6SS-accessory protein, is abundantly localized in the OM. It interacts with the IM proteins PpkA and TssM so that the T6SS can be formed across the cells. Without TssW, PpkA and the inner membrane complex foci were primarily localized to the cell pole, significantly impairing the T6SS functions. PpkA can activate T6SS assembly through phosphorylation of its binding partner Fha, which subsequently triggers membrane complex-baseplate assembly. PpkA itself interacts with both T6SS membrane proteins and baseplate proteins and undergoes LLPS, similar to Fha. Through recruiting multiple T6SS structural components, this TssW-PpkA-Fha axis controls the location and initiation of the T6SS assembly.

Our observations that varying the levels of TssW, PpkA, and Fha affects the number and positioning of T6SS assemblies suggest a stoichiometric balance is likely important for optimal T6SS assembly and firing control. Similar effects have been reported for other T6SS components. For instance, altering the ratio of TssA-TagA proteins modulates sheath-formation kinetics in *Vibrio cholerae*, and the balance between TssA and TssM impacts activation of the *Vibrio fischeri* T6SS2 (42, 43). To estimate the number of molecules within Fha and PpkA foci in AAC00-1, we employed a LacI*-lacO*-based calibration system. We constructed *V. cholerae* V52 strains carrying 3, 6, or 13 *lacO* repeats integrated at the *lacZ* locus, as previously described (43). Expression of LacI^mut^_sfGFP in these strains yielded fluorescent foci with expected signal intensities corresponding to 6, 12, and 26 sfGFP copies, respectively, allowing us to generate a standard curve for quantification. Comparing these reference values to the fluorescence intensity of Fha_sfGFP and sfGFP_PpkA foci, we estimated that individual Fha foci contain ~60 to 600 molecules, whereas PpkA foci contain ~20 to 80 molecules (Fig. S5J and K). These measurements provide quantitative insight into the architecture of T6SS initiation sites. Although PpkA is known to regulate T6SS activities post-translationally in many strains, including *P. aeruginosa*, *Serratia marcescens*, and *Agrobacterium tumefaciens*, the detailed mechanism of how it affects the assembly of T6SS MC and BP is elusive. In *A. tumefaciens*, PpkA-mediated phosphorylation of TssL is required for TssL-Fha interaction and contributes to TssM conformational change, but whether TssL and TssM can assemble the MC independently of Fha remains unclear (31). Our findings highlight that PpkA not only serves as a regulatory factor but also functions as a critical T6SS structural component. Although the interaction between PpkA and Fha is independent of TssW (Fig. 4E), it is unclear whether TssW modulates the efficiency of this interaction or merely serves as a spatial anchor. Further investigations utilizing structural biology techniques are required to determine the precise spatial relationships among PpkA, Fha, TssW, and the conserved core components of the T6SS.

LLPS refers to the phenomenon of macromolecules separating into a dilute phase and a much higher concentrated condensate as a membraneless compartment. Known LLPS-forming bacterial proteins include ribonuclease E, RNA polymerase, scaffold protein PodJ, transcription termination factor Rho, and the membrane-bound ABC transporter Rv1747 (44–48). These LLPS condensates are found in distantly related species and are associated with diverse intracellular functions. By modulating the T6SS activities, LLPS can extend its influence to extracellular species and promote bacterial fitness. The condensates formed by PpkA and Fha may offer a list of potential and nonexclusive benefits, including bypassing the diffusion law to facilitate protein assembly, excluding irrelevant proteins to increase efficiency, detaching binding partners between structural and chaperone proteins, preventing wrongly formed protein interactions, stabilizing a polymerizing T6SS apparatus, and inhibiting excessive T6SS structure assembly.

Although the LLPS properties are clearly demonstrated in those known cases, the contribution of LLPS to biological functions remains largely elusive. One challenge is the intrinsically disordered region, or other LLPS-required domains may be multifunctional, i.e., they may be involved in not only forming the condensates but also interacting with client proteins. Inhibiting LLPS formation through domain truncations or through chemical treatment of 1,6-hexanediol may result in unwanted pleiotropic effects. Interestingly, we noticed that the transmembrane domain of PpkA may be required for forming LLPS *in vivo* as the sfGFP_PpkA^∆TMD^ construct failed to form foci in cells (Fig. S5L), and deletion of the transmembrane domain abolished the T6SS assembly and T6SS-mediated killing activities (Fig. S5M through O). Additionally, plasmid-borne PpkA^∆TMD^ can complement the *tssW* deletion (Fig. 3E through G; Fig. S3A and B), suggesting it is kinase-active and capable of interacting with other T6SS proteins. These results collectively provide strong evidence supporting that LLPS is crucial for PpkA-mediated T6SS functions. Because the PpkA^∆TMD^ can form condensates *in vitro*, the transmembrane domain may promote PpkA LLPS formation by sequestering PpkA to the membrane and creating a locally concentrated condensate.

The mechanisms driving polar localization of PpkA-Fha condensates remain unclear. Potential contributors include nucleoid exclusion, preferential binding to polar lipids (e.g., cardiolipin), or interactions with polar scaffold proteins (49–51). Future studies are needed to dissect the relative contributions of these factors. Notably, the evenly distributed OM protein TssW, rather than the conserved T6SS OM component TssJ, serves as a critical anchor for non-polar T6SS assembly.

Finally, the T6SSs have displayed diverse functions, which can be explored for controlling multidrug-resistant pathogens *in vivo* or in the natural environment. The T6SS in *A. citrulli* is a very potent system that can penetrate the cell envelope of diverse cell types, including pathogenic *Candida* fungal pathogens that have been considered in recent years as a global public health threat (11, 52, 53). How the *A. citrulli* T6SS acquires such superior killing capabilities is unclear, but its potential as a valuable model for studying T6SS functions and as a much-needed tool to treat drug-resistant pathogens is evident. Like other T6SSs, the functions of the *A. citrulli* T6SS have been largely attributed to its large effector arsenal (11). Here, our results on TssW-PpkA-Fha reveal another layer of regulation by controlling its localization and assembly efficiency. Because T6SS activity is primarily contact-dependent, its spatial positioning is crucial. For example, in *P. aeruginosa*, a “Tit-for-Tat” response enables rapid assembly of a T6SS at the precise site of an incoming attack, ensuring accurate retaliation (27). In *S. marcescens*, loss of the PppA phosphatase impairs redistribution of the T6SS and markedly diminishes antibacterial activity despite normal Hcp secretion (30). We show that the polar T6SS assembly reduced killing, likely resulting from less frequent contact with neighboring cells, further supporting that T6SS positioning is crucial to competitive fitness. These findings may offer a conceptual basis for future efforts aimed at engineering synthetic T6SSs with tunable spatial dynamics or activity. Reconstitution of the *A. citrulli* T6SS in heterologous or commensal strains could, in principle, enable targeted interference with competing microorganisms. Furthermore, incorporating the TssW-PpkA-Fha regulatory module into T6SS systems that naturally lack such components may help modulate their localization or firing behavior. Future cryo-ET *in situ* imaging analysis is required to image the interaction of the accessory complex with the conserved complex. These features may further advance our understanding of T6SS assembly and guide the rational design of synthetic T6SSs for biotechnological and clinical applications.

## MATERIALS AND METHODS

### Bacterial strains and growth conditions

Strains were routinely grown in LB (lysogeny broth), 7H9, 7H10, or yeast extract–peptone–dextrose (YPD) media following standard culturing conditions. Antibiotics were incorporated at the specified concentrations: kanamycin (25 µg/mL), gentamicin (10 µg/mL), and carbenicillin (50 µg/mL). Plasmids, strains, and primers employed in this study are available in Table S1.

### Plasmids and mutants’ construction

Plasmids were assembled via Gibson assembly and subsequently verified through Sanger sequencing. All of the *A. citrulli* AAC00-1 mutants were constructed via homologous recombination, employing the pEXG2.0 suicide vector (54). AAC00-1 strains were mixed with *E. coli* WM6026, which carried pEXG2.0 plasmids featuring homologous arms. This mixture was then incubated at 37°C for approximately 6 h. Transconjugants were subsequently screened on LB agar plates with gentamicin. After purification, the transconjugants were inoculated into liquid LB media and cultivated at 28°C for 24 h. Following this, subculture was carried out in fivefold diluted liquid LB media supplemented with 10% sucrose for an additional 24 h. The resulting cultures were spread on LB agar plates, and colonies that exhibited sucrose resistance were confirmed via PCR.

### Bacterial competition assay

AAC00-1 killer strains were cultured in liquid LB media until reaching an OD_600_ of approximately 2. In parallel, *E. coli* prey cells, sourced from overnight-cultured LB agar plates, *C. albicans* and *C. auris* from YPD agar plates, and *M. smegmatis* from 7H9 broth cultured for 2 days, were prepared. Killer cells were mixed with *E. coli* prey at a 10:1 ratio and with *Candida* prey cells at the same ratio, then spotted onto LB agar and YPD agar plates, respectively, followed by incubation for 3 h at 37°C for *E. coli* and 20 h at 37°C for *Candida*. Similarly, killer and *M. smegmatis* prey cells were mixed at a 5:1 ratio and spotted onto 7H10 plates, incubated for 20 h at 37°C. Subsequently, the survival of both killer and prey cells was assessed through 10-fold serial dilution on selective media. Error bars denote the mean ± standard deviation, calculated from a minimum of three independent biological replicates. *P*-values were determined by performing a one-way analysis of variance with Dunnett’s multiple comparisons test, using GraphPad Prism software (version 10.4.1).

### Western blotting analysis

The Western blotting analyses were performed as described previously (11). In brief, proteins of interest were electrophoresed on SDS-PAGE gels (Yeasen Biotechnology) and subsequently transferred onto polyvinylidene fluorid (PVDF) membranes (Bio-Rad). The membranes were then subjected to a 1 h treatment with a blocking buffer (5% [wt/vol] non-fat milk, 50 mM Tris, 150 mM NaCl, 0.1% [vol/vol] Tween-20, pH 7.6) at room temperature. Following this, the membranes underwent sequential incubation with primary antibodies and secondary horseradish peroxidase (HRP)-conjugated antibodies, with all incubations occurring in an antibody diluent buffer (1% [wt/vol] non-fat milk, 50 mM Tris, 150 mM NaCl, 0.1% [vol/vol] Tween-20, pH 7.6). Signal detection was achieved using Clarity ECL solution (Bio-Rad). Monoclonal antibodies directed against epitope tags were procured from Smart-lifesciences (FLAG, Product # SLAB0102; 6His, Product # SLAB2803), ABclonal (V5, Product # AE017), ABmart (OmpF, Product # PH5808S), and Biolegend (RpoB, Product # 663905). Custom-made polyclonal antibodies targeting Aave_1465 (Hcp) and Aave_1468 (Fha) were provided by Shanghai Youlong Biotech. HRP-linked secondary antibodies were obtained from ZSGB-Bio (Product # ZB-2305 [mouse] and # ZB-2301 [rabbit]). The experiments were done at least three times, and a representative result is shown.

### Protein secretion assay

Cultures were aerobically cultivated in liquid LB media at 28°C overnight, reaching an OD_600_ of approximately 2. These cultures were subsequently normalized to OD_600_ = 2. For the “Culture” samples, 40 µL of the normalized cultures were extracted and mixed with 10 µL of 5× SDS loading buffer (Epizyme Biotech). The remaining cultures were subsequently centrifuged at 10,000 × *g* for 2 min, resulting in the separation of supernatants, which were labeled as the “Sec” samples, and pelleted cells. The pelleted cells were resuspended in fresh liquid LB media and used as the “Cell” samples. All “Culture,” “Cell,” and “Sec” samples were subjected to analysis by Western blotting. The experiments were done at least three times, and a representative result is shown.

### Protein pull-down assays

Genes of interest were cloned with epitope tags and inserted into pET, pBAD, and pBBR vectors, as specified. For pET vectors, *E. coli* BL21(DE3) cells carrying the relevant plasmids were cultured in LB media with appropriate antibiotics until reaching an OD_600_ of approximately 0.6. Induction was carried out by adding 1 mM isopropyl-β-D-thiogalactopyranoside (IPTG) and allowing overnight growth at 20°C. For pBAD vectors, *E. coli* T-Fast cells carrying the relevant plasmids were cultured in LB media with appropriate antibiotics until they reached an OD_600_ of approximately 0.6. Induction was carried out by adding 0.1% L-arabinose and incubating for 3 h at 30°C. In the case of pBBR vectors, *E. coli* T-Fast cells harboring the corresponding plasmids were grown in LB media with the necessary antibiotics for approximately 6 h at 37°C. Following growth, the cells were harvested via centrifugation, resuspended in lysis buffer (20 mM Tris, 500 mM NaCl, 50 mM imidazole, pH 8.0, 1× protease inhibitor [Thermo Scientific]), and lysed by sonication on ice. After centrifugation, the resulting supernatants were mixed as specified and loaded onto Ni-NTA resin (Smart-lifesciences). The mixtures were rotated at 4°C for 1 h, washed four times with wash buffer (20 mM Tris, 500 mM NaCl, 50 mM imidazole, pH 8.0), and eluted using elution buffer (20 mM Tris, 500 mM NaCl, 400 mM imidazole, pH 8.0). The input and eluted samples were analyzed by Western blotting. The experiments were done at least three times, and a representative result is shown.

### PhoA alkaline phosphatase assay

The N- or C- terminus of various proteins was fused to alkaline phosphatase (PhoA) or its variants as indicated, and the resulting constructs were expressed using either the pBBR vector (for *A. citrulli* AAC00-1) or the pBAD vector (for *E. coli* DH5α). *A. citrulli* AAC00-1 strains harboring the respective plasmids were cultured aerobically in LB broth at 28°C until reaching an OD_600_ of approximately 2. *E. coli* DH5α strains harboring the respective plasmids were cultured aerobically in LB broth at 37°C until reaching an OD_600_ of approximately 1. Cultures were then spotted onto LB agar plates supplemented with appropriate antibiotics, BCIP (80  µg/mL), and 0.01% L-arabinose (for induction from the pBAD vector in *E. coli* strains). Plates were incubated at 37°C for at least 16 h prior to imaging. The experiments were done at least two times, and a representative result is shown.

### Cell fractionation

Cultures were aerobically cultivated in liquid LB media at 28°C overnight, reaching an OD_600_ of approximately 2. Cells were harvested by centrifugation at 4,500 × *g* for 10 min at 4°C, resuspended in 10 mM HEPES (pH 7.4), and lysed using a French press. The lysate was cleared by centrifugation at 5,000 × *g* for 20 min, filtered through a 0.22 µm filter, and ultracentrifuged at 57,000 × *g* for 40 min at 4°C to collect the total membrane fraction. The total membrane fraction was applied to an eight-step sucrose density gradient (30%, 35%, 40%, 45%, 50%, 55%, 60%, and 65%) in 10 mM HEPES (pH 7.4). The gradient was subjected to ultracentrifugation at 197,000 × *g* for 24 h at 4°C. Fractions were collected, boiled at 98°C for 10 min, and resolved by SDS-PAGE for Western blotting analysis. NADH oxidase activity was measured in a 96-well plate, with each fraction diluted to 20 µL in 180 µL reaction buffer (50 mM Tris, pH 7.5, 0.2 mM DTT, 0.5 mM NADH). Absorbance was measured at 340 nm after 1 h of incubation at room temperature. Each fraction was tested in duplicate.

### *In vitro* LLPS assay

Purified proteins were first dialyzed against an LLPS buffer (10 mM Na_2_HPO_4_/NaH_2_PO_4_ and 200 mM NaCl, pH 7.4). After dialysis, proteins were concentrated to approximately 80 µM using centrifugal filters with Ultracel-30K membranes. For imaging preparations, the concentrated protein solutions underwent a twofold serial dilution in LLPS buffer. Equal volumes of each diluted protein sample were then mixed with 10% dextran-70 in LLPS buffer. These mixtures were placed onto glass slides for microscopy. Imaging was performed using a ZEISS LSM 980 with Airyscan 2, utilizing a 63×/1.40 oil immersion objective and a 488 nm laser. The experiments were repeated at least twice, and a typical example of the results is presented.

### Fluorescence microscopy of T6SS assembly

Cells were grown overnight in liquid LB media to OD_600_ ~ 2 at 28°C. The cultures were then centrifuged at 4,500 × *g* for 2 min, resuspended in liquid LB media to OD_600_ ~ 10, and spotted onto 1% agarose-0.5 × phosphate-buffered saline (PBS) pads. Wide-field fluorescence imaging was performed using a Nikon Ti2-E inverted microscope equipped with the Perfect Focus System and a Plan Apo 100× Oil Ph3 DM objective lens (NA 1.45). Fluorescence excitation was achieved using ET-GFP (Chroma #49002) and ET-mCherry (Chroma #49008) filter sets. For high-resolution imaging in Fig. 2A, 3D-SIM was performed using a Nikon Ti-E A1R HD25+ SIM system equipped with a Perfect Focus System, a CFI SR HP Apochromat TIRF 100× oil objective lens (NA 1.49), and 488 nm and 561 nm excitation lasers. For analysis of TssL localization, fluorescence and phase-contrast images were overlaid, and the subcellular position of TssL foci was determined relative to cell boundaries to distinguish polar and non-polar foci. For TssB dynamics, time-lapse imaging was performed at 10 second intervals for a total duration of 5  min to monitor the assembly and disassembly of TssB foci. The persistence time of individual TssB foci was defined as the time between their initial appearance and disappearance. All imaging experiments were done at least two times, and a representative result is shown.

### FRAP

Droplets containing sfGFP-tagged PpkA or PpkA^∆TMD^ proteins mixed with dextran-70 were placed on glass slides for imaging. Images were captured using a ZEISS LSM 980 Airyscan 2 microscope with a 63×/1.40 oil immersion objective and a 488 nm laser. Photobleaching was carried out by exposing selected regions to the laser at 100% power for 10 ms. The recovery of fluorescence post-bleaching was monitored by comparing the intensity in the bleached area against an adjacent non-bleached region, using the “FRAP_profiler_V2” plugin in Fiji software. The intensity before bleaching was set as 100% reference, and the fluorescence recovery was calculated at subsequent time intervals. All imaging experiments were done two times, and a representative result is shown.

### Plasmolysis assays

Cells were grown overnight in liquid LB media to OD_600_ ~2 at 28°C. The cultures were then centrifuged at 4,500 × *g* for 2 min, resuspended in liquid LB media or 3 M sorbitol, and spotted onto 1% agarose-0.5 × PBS pads or 1% agarose-3 M sorbitol pads, respectively. Image acquisition was performed using the Nikon Ti-E A1R HD25+ SIM S microscope, which was equipped with the Perfect Focus System and CFI SR HP Apochromat TIRF 100XC (NA 1.49) objective lens, along with the 488 and 561  nm excitation lasers. The 3D-SIM imaging was conducted. The imaging experiment was done two times, and a representative result is shown.

### Fluorescence quantification

Experiments were performed as previously described with minor modifications (43). *lacO* arrays were designed to contain repeats of the operator sequence interspersed with 18 random nucleotide spacers. LacI^mut^, which lacks the C-terminal tetramerization domain but retains the ability to bind DNA similarly to the wild-type LacI, was fused to sfGFP and used to estimate the number of sfGFP molecules required to detect fluorescent foci of specific intensity in cells. LacI^mut^_sfGFP was expressed from the pBAD24 plasmid and induced with 0.002% L-arabinose. Raw sfGFP signals from LacI, Fha, and PpkA foci were collected using Fiji. All measurements were performed on unprocessed images.

### RNA extraction and qRT-PCR analysis

AAC00-1 strains were cultured in LB medium to OD_600_ ~2. Cells were harvested by centrifugation and resuspended in 800  µL of lysis buffer (1% [wt/vol] SDS, 2  mM EDTA) by vortex for 1 min. An equal volume (800  µL) of pre-warmed acidic phenol (65°C) was immediately added, and the mixture was gently inverted to mix. The tubes were incubated at 65°C for 5 min, with brief mixing every minute, and then placed on ice for 10 min. After centrifugation at 13,000  ×  *g* for 2 min, the upper aqueous phase was carefully transferred to a new tube. An equal volume of absolute ethanol was added, and total RNA was purified using the RNAprep Pure Cell/Bacteria Kit (TIANGEN, #DP430), following the manufacturer’s instructions. First-strand complementary DNA was synthesized from the purified RNA using a reverse transcription kit (Vazyme, R333-00). qRT-PCR was performed with SYBR Green Master Mix (Vazyme, Q312-02) on a BIO-GENER Q9604 real-time PCR system. Gene expression levels were normalized to 16S rRNA, and relative transcript levels were calculated using the 2^−ΔΔCt^ method. Each sample was measured in triplicate and repeated at least three times.

### Protein purification

Proteins tagged with Strep-tagII were produced in *E. coli* BL21(DE3) using pET vectors. Cultures were grown in LB broth until OD_600_ ~0.6 at 37°C and were then induced with 1 mM IPTG at 16°C for 16 h. Cells were harvested by centrifugation at 4,500 × *g* for 15 min at 4°C. The cell pellets were resuspended in PBS (10 mM Na_2_HPO_4_, 140 mM NaCl, 3 mM KCl, pH 7.4) and subjected to sonication for lysis. The lysed cells were centrifuged at 15,000 × *g* for 30 min to separate the supernatant. The clear supernatant was incubated with Streptactin beads (Smart‐lifesciences) for affinity chromatography. Bound proteins were eluted using PBS buffer supplemented with 2.5 mM biotin.

### Bioinformatic analysis

All gene sequences of *A. citrulli* AAC00-1 were retrieved from the draft genome assembly (GenBank NC_008752.1), and managed and analyzed by Benchling. PpkA and TssW sequences were analyzed with the PHMMER tool of HMMER webserver. Sequence alignments of TssM were performed using Clustal Omega and phylogeny (bootstrap = 1,000) was generated by the Interactive Tree Of Life server with default settings (55, 56).

## Data Availability

Data supporting the findings of this study are available within the paper or from the corresponding author upon request.

## SUPPLEMENTAL MATERIAL

Data S1 mbio.01879-25-s0001.txt Sequence file of TssM homologs for Fig. 1H.

Supplemental Material mbio.01879-25-s0002.pdf Supplemental figures, table, and Data S1 caption.

Movie S1 mbio.01879-25-s0003.mp4 Time-lapse imaging showing the subcellular localization of chromosomally encoded sfGFP_TssL and TssB_mCherry2 in AAC00-1 WT and Δ*tssW*.

Movie S2 mbio.01879-25-s0004.mp4 Time-lapse imaging showing the subcellular localization of chromosomally encoded sfGFP_TssL and TssB_mCherry2 in AAC00-1 Δ*tssW* expressing PpkA, PpkA^Kinase^, or PpkA^ΔTMD^.

Movie S3 mbio.01879-25-s0005.mp4 Time-lapse imaging showing the subcellular localization of chromosomally encoded sfGFP_TssL and TssB_mCherry2 in AAC00-1 carrying the empty pBBR plasmid.

Movie S4 mbio.01879-25-s0006.mp4 Time-lapse imaging showing the subcellular localization of chromosomally encoded sfGFP_TssL and TssB_mCherry2 in AAC00-1 expressing Fha.

Movie S5 mbio.01879-25-s0007.mp4 Time-lapse imaging showing the subcellular localization of chromosomally encoded sfGFP_TssL and TssB_mCherry2 in AAC00-1 expressing PpkA.

Movie S6 mbio.01879-25-s0008.mp4 Time-lapse imaging showing the subcellular localization of chromosomally encoded sfGFP_TssL and TssB_mCherry2 in AAC00-1 expressing PpkA and TssW.

Movie S7 mbio.01879-25-s0009.mp4 Time-lapse imaging showing the subcellular localization of chromosomally encoded sfGFP_TssL and TssB_mCherry2 in AAC00-1 expressing PpkA and Fha.

ASM does not own the copyrights to Supplemental Material that may be linked to, or accessed through, an article. The authors have granted ASM a non-exclusive, world-wide license to publish the Supplemental Material files. Please contact the corresponding author directly for reuse.

## References

[1] Costa TRD, Felisberto-Rodrigues C, Meir A, Prevost MS, Redzej A, Trokter M, Waksman G. 2015. Secretion systems in Gram-negative bacteria: structural and mechanistic insights. Nat Rev Microbiol 13:343–359. doi:10.1038/nrmicro3456 25978706

[2] Subramanian S, Kearns DB. 2019. Functional regulators of bacterial flagella. Annu Rev Microbiol 73:225–246. doi:10.1146/annurev-micro-020518-115725 31136265 PMC7110939

[3] Galán JE, Waksman G. 2018. Protein-injection machines in bacteria. Cell 172:1306–1318. doi:10.1016/j.cell.2018.01.034 29522749 PMC5849082

[4] Bisson-Filho AW, Hsu Y-P, Squyres GR, Kuru E, Wu F, Jukes C, Sun Y, Dekker C, Holden S, VanNieuwenhze MS, Brun YV, Garner EC. 2017. Treadmilling by FtsZ filaments drives peptidoglycan synthesis and bacterial cell division. Science 355:739–743. doi:10.1126/science.aak9973 28209898 PMC5485650

[5] Ho BT, Dong TG, Mekalanos JJ. 2014. A view to a kill: the bacterial type VI secretion system. Cell Host Microbe 15:9–21. doi:10.1016/j.chom.2013.11.008 24332978 PMC3936019

[6] Pukatzki S, Ma AT, Sturtevant D, Krastins B, Sarracino D, Nelson WC, Heidelberg JF, Mekalanos JJ. 2006. Identification of a conserved bacterial protein secretion system in Vibrio cholerae using the Dictyostelium host model system. Proc Natl Acad Sci USA 103:1528–1533. doi:10.1073/pnas.0510322103 16432199 PMC1345711

[7] Hood RD, Singh P, Hsu F, Güvener T, Carl MA, Trinidad RRS, Silverman JM, Ohlson BB, Hicks KG, Plemel RL, Li M, Schwarz S, Wang WY, Merz AJ, Goodlett DR, Mougous JD. 2010. A type VI secretion system of Pseudomonas aeruginosa targets a toxin to bacteria. Cell Host Microbe 7:25–37. doi:10.1016/j.chom.2009.12.007 20114026 PMC2831478

[8] Trunk K, Peltier J, Liu Y-C, Dill BD, Walker L, Gow NAR, Stark MJR, Quinn J, Strahl H, Trost M, Coulthurst SJ. 2018. The type VI secretion system deploys antifungal effectors against microbial competitors. Nat Microbiol 3:920–931. doi:10.1038/s41564-018-0191-x 30038307 PMC6071859

[9] Le NH, Pinedo V, Lopez J, Cava F, Feldman MF. 2021. Killing of Gram-negative and Gram-positive bacteria by a bifunctional cell wall-targeting T6SS effector. Proc Natl Acad Sci USA 118:6–11. doi:10.1073/pnas.2106555118 PMC8501793 34588306

[10] Mougous JD, Cuff ME, Raunser S, Shen A, Zhou M, Gifford CA, Goodman AL, Joachimiak G, Ordoñez CL, Lory S, Walz T, Joachimiak A, Mekalanos JJ. 2006. A virulence locus of Pseudomonas aeruginosa encodes a protein secretion apparatus. Science 312:1526–1530. doi:10.1126/science.1128393 16763151 PMC2800167

[11] Pei T-T, Kan Y, Wang Z-H, Tang M-X, Li H, Yan S, Cui Y, Zheng H-Y, Luo H, Liang X, Dong T. 2022. Delivery of an Rhs-family nuclease effector reveals direct penetration of the gram-positive cell envelope by a type VI secretion system in Acidovorax citrulli. mLife 1:66–78. doi:10.1002/mlf2.12007 38818323 PMC10989746

[12] Wang J, Brodmann M, Basler M. 2019. Assembly and subcellular localization of bacterial type VI secretion systems. Annu Rev Microbiol 73:621–638. doi:10.1146/annurev-micro-020518-115420 31226022

[13] Durand E, Nguyen VS, Zoued A, Logger L, Péhau-Arnaudet G, Aschtgen M-S, Spinelli S, Desmyter A, Bardiaux B, Dujeancourt A, Roussel A, Cambillau C, Cascales E, Fronzes R. 2015. Biogenesis and structure of a type VI secretion membrane core complex. Nature 523:555–560. doi:10.1038/nature14667 26200339

[14] Shneider MM, Buth SA, Ho BT, Basler M, Mekalanos JJ, Leiman PG. 2013. PAAR-repeat proteins sharpen and diversify the type VI secretion system spike. Nature 500:350–353. doi:10.1038/nature12453 23925114 PMC3792578

[15] Brunet YR, Zoued A, Boyer F, Douzi B, Cascales E. 2015. The type VI secretion TssEFGK-VgrG phage-like baseplate is recruited to the TssJLM membrane complex via multiple contacts and serves as assembly platform for tail tube/sheath polymerization. PLoS Genet 11:e1005545. doi:10.1371/journal.pgen.1005545 26460929 PMC4604203

[16] Basler M, Pilhofer M, Henderson GP, Jensen GJ, Mekalanos JJ. 2012. Type VI secretion requires a dynamic contractile phage tail-like structure. Nature 483:182–186. doi:10.1038/nature10846 22367545 PMC3527127

[17] Zoued A, Durand E, Brunet YR, Spinelli S, Douzi B, Guzzo M, Flaugnatti N, Legrand P, Journet L, Fronzes R, Mignot T, Cambillau C, Cascales E. 2016. Priming and polymerization of a bacterial contractile tail structure. Nature 531:59–63. doi:10.1038/nature17182 26909579

[18] Mougous JD, Gifford CA, Ramsdell TL, Mekalanos JJ. 2007. Threonine phosphorylation post-translationally regulates protein secretion in Pseudomonas aeruginosa. Nat Cell Biol 9:797–803. doi:10.1038/ncb1605 17558395

[19] Bönemann G, Pietrosiuk A, Diemand A, Zentgraf H, Mogk A. 2009. Remodelling of VipA/VipB tubules by ClpV-mediated threading is crucial for type VI protein secretion. EMBO J 28:315–325. doi:10.1038/emboj.2008.269 19131969 PMC2646146

[20] Allsopp LP, Bernal P. 2023. Killing in the name of: T6SS structure and effector diversity. Microbiology (Reading) 169:001367. doi:10.1099/mic.0.001367 37490402 PMC10433429

[21] Russell AB, Peterson SB, Mougous JD. 2014. Type VI secretion system effectors: poisons with a purpose. Nat Rev Microbiol 12:137–148. doi:10.1038/nrmicro3185 24384601 PMC4256078

[22] Dong TG, Ho BT, Yoder-Himes DR, Mekalanos JJ. 2013. Identification of T6SS-dependent effector and immunity proteins by Tn-seq in Vibrio cholerae. Proc Natl Acad Sci USA 110:2623–2628. doi:10.1073/pnas.1222783110 23362380 PMC3574944

[23] Pukatzki S, Ma AT, Revel AT, Sturtevant D, Mekalanos JJ. 2007. Type VI secretion system translocates a phage tail spike-like protein into target cells where it cross-links actin. Proc Natl Acad Sci USA 104:15508–15513. doi:10.1073/pnas.0706532104 17873062 PMC2000545

[24] Monjarás Feria J, Valvano MA. 2020. An overview of anti-eukaryotic T6SS effectors. Front Cell Infect Microbiol 10:584751. doi:10.3389/fcimb.2020.584751 33194822 PMC7641602

[25] Smith WPJ, Brodmann M, Unterweger D, Davit Y, Comstock LE, Basler M, Foster KR. 2020. The evolution of tit-for-tat in bacteria via the type VI secretion system. Nat Commun 11:5395. doi:10.1038/s41467-020-19017-z 33106492 PMC7589516

[26] Wong MJQ, Liang X, Smart M, Tang L, Moore R, Ingalls B, Dong TG. 2016. Microbial herd protection mediated by antagonistic interaction in polymicrobial communities. Appl Environ Microbiol 82:6881–6888. doi:10.1128/AEM.02210-16 27637882 PMC5103087

[27] Basler M, Ho BT, Mekalanos JJ. 2013. Tit-for-tat: type VI secretion system counterattack during bacterial cell-cell interactions. Cell 152:884–894. doi:10.1016/j.cell.2013.01.042 23415234 PMC3616380

[28] Ho BT, Basler M, Mekalanos JJ. 2013. Type 6 secretion system-mediated immunity to type 4 secretion system-mediated gene transfer. Science 342:250–253. doi:10.1126/science.1243745 24115441 PMC4034461

[29] Wilton M, Wong MJQ, Tang L, Liang X, Moore R, Parkins MD, Lewenza S, Dong TG. 2016. Chelation of membrane-bound cations by extracellular DNA activates the type VI secretion system in Pseudomonas aeruginosa. Infect Immun 84:2355–2361. doi:10.1128/IAI.00233-16 27271742 PMC4962633

[30] Ostrowski A, Cianfanelli FR, Porter M, Mariano G, Peltier J, Wong JJ, Swedlow JR, Trost M, Coulthurst SJ. 2018. Killing with proficiency: integrated post-translational regulation of an offensive Type VI secretion system. PLoS Pathog 14:e1007230. doi:10.1371/journal.ppat.1007230 30052683 PMC6082577

[31] Lin J-S, Wu H-H, Hsu P-H, Ma L-S, Pang Y-Y, Tsai M-D, Lai E-M. 2014. Fha interaction with phosphothreonine of TssL activates type VI secretion in Agrobacterium tumefaciens. PLoS Pathog 10:e1003991. doi:10.1371/journal.ppat.1003991 24626341 PMC3953482

[32] Yang Z, Zhou X, Ma Y, Zhou M, Waldor MK, Zhang Y, Wang Q. 2018. Serine/threonine kinase PpkA coordinates the interplay between T6SS2 activation and quorum sensing in the marine pathogen Vibrio alginolyticus. Environ Microbiol 20:903–919. doi:10.1111/1462-2920.14039 29314504 PMC6452854

[33] Bingle LE, Bailey CM, Pallen MJ. 2008. Type VI secretion: a beginner’s guide. Curr Opin Microbiol 11:3–8. doi:10.1016/j.mib.2008.01.006 18289922

[34] Hoffman CS, Wright A. 1985. Fusions of secreted proteins to alkaline phosphatase: an approach for studying protein secretion. Proc Natl Acad Sci USA 82:5107–5111. doi:10.1073/pnas.82.15.5107 3860846 PMC390508

[35] Manoil C, Mekalanos JJ, Beckwith J. 1990. Alkaline phosphatase fusions: sensors of subcellular location. J Bacteriol 172:515–518. doi:10.1128/jb.172.2.515-518.1990 2404939 PMC208471

[36] Rojas ER, Billings G, Odermatt PD, Auer GK, Zhu L, Miguel A, Chang F, Weibel DB, Theriot JA, Huang KC. 2018. The outer membrane is an essential load-bearing element in Gram-negative bacteria. Nature 559:617–621. doi:10.1038/s41586-018-0344-3 30022160 PMC6089221

[37] Cardoso Mendes Moura EC, Polissi A, Sperandeo P. 2022. Membrane fractionation by isopycnic sucrose density gradient centrifugation for qualitative analysis of LPS in Escherichia coli. Methods Mol Biol 2548:53–69. doi:10.1007/978-1-0716-2581-1_4 36151491

[38] Pei T-T, Wang X-Y, Zhang Y-Q, An Y, Luo H, Kan Y, Li H, Tang M-X, Ye Z-Y, Liang J-X, Jian T, Zheng H-Y, Wang Z-H, Liang X, Liu X, Zhang M, Dong T. 2025. Fha initiates the inside-out assembly of the type VI secretion system. Cell Rep 44:115990. doi:10.1016/j.celrep.2025.115990 40650907

[39] Ellis RJ. 2001. Macromolecular crowding: an important but neglected aspect of the intracellular environment. Curr Opin Struct Biol 11:114–119. doi:10.1016/s0959-440x(00)00172-x 11179900

[40] Russell AB, Wexler AG, Harding BN, Whitney JC, Bohn AJ, Goo YA, Tran BQ, Barry NA, Zheng H, Peterson SB, Chou S, Gonen T, Goodlett DR, Goodman AL, Mougous JD. 2014. A type VI secretion-related pathway in Bacteroidetes mediates interbacterial antagonism. Cell Host Microbe 16:227–236. doi:10.1016/j.chom.2014.07.007 25070807 PMC4136423

[41] Coyne MJ, Roelofs KG, Comstock LE. 2016. Type VI secretion systems of human gut Bacteroidales segregate into three genetic architectures, two of which are contained on mobile genetic elements. BMC Genomics 17:58. doi:10.1186/s12864-016-2377-z 26768901 PMC4714493

[42] Smith S, Salvato F, Garikipati A, Kleiner M, Septer AN. 2021. Activation of the type VI secretion system in the squid symbiont Vibrio fischeri requires the transcriptional regulator tasr and the structural proteins TssM and TssA. J Bacteriol 203:e0039921. doi:10.1128/JB.00399-21 34370559 PMC8508121

[43] Schneider JP, Nazarov S, Adaixo R, Liuzzo M, Ringel PD, Stahlberg H, Basler M. 2019. Diverse roles of TssA‐like proteins in the assembly of bacterial type VI secretion systems. EMBO J 38:e100825. doi:10.15252/embj.2018100825 31403721 PMC6745524

[44] Al-Husini N, Tomares DT, Bitar O, Childers WS, Schrader JM. 2018. α-Proteobacterial RNA degradosomes assemble liquid-liquid phase-separated RNP bodies. Mol Cell 71:1027–1039. doi:10.1016/j.molcel.2018.08.003 30197298 PMC6151146

[45] Ladouceur A-M, Parmar BS, Biedzinski S, Wall J, Tope SG, Cohn D, Kim A, Soubry N, Reyes-Lamothe R, Weber SC. 2020. Clusters of bacterial RNA polymerase are biomolecular condensates that assemble through liquid-liquid phase separation. Proc Natl Acad Sci USA 117:18540–18549. doi:10.1073/pnas.2005019117 32675239 PMC7414142

[46] Heinkel F, Abraham L, Ko M, Chao J, Bach H, Hui LT, Li H, Zhu M, Ling YM, Rogalski JC, Scurll J, Bui JM, Mayor T, Gold MR, Chou KC, Av-Gay Y, McIntosh LP, Gsponer J. 2019. Phase separation and clustering of an ABC transporter in Mycobacterium tuberculosis. Proc Natl Acad Sci USA 116:16326–16331. doi:10.1073/pnas.1820683116 31366629 PMC6697873

[47] Krypotou E, Townsend GE, Gao X, Tachiyama S, Liu J, Pokorzynski ND, Goodman AL, Groisman EA. 2023. Bacteria require phase separation for fitness in the mammalian gut. Science 379:1149–1156. doi:10.1126/science.abn7229 36927025 PMC10148683

[48] Tan W, Cheng S, Li Y, Li X-Y, Lu N, Sun J, Tang G, Yang Y, Cai K, Li X, Ou X, Gao X, Zhao G-P, Childers WS, Zhao W. 2022. Phase separation modulates the assembly and dynamics of a polarity-related scaffold-signaling hub. Nat Commun 13:7181. doi:10.1038/s41467-022-35000-2 36418326 PMC9684454

[49] Lasker K, Boeynaems S, Lam V, Scholl D, Stainton E, Briner A, Jacquemyn M, Daelemans D, Deniz A, Villa E, Holehouse AS, Gitler AD, Shapiro L. 2022. The material properties of a bacterial-derived biomolecular condensate tune biological function in natural and synthetic systems. Nat Commun 13:5643. doi:10.1038/s41467-022-33221-z 36163138 PMC9512792

[50] Surovtsev IV, Jacobs-Wagner C. 2018. Subcellular organization: a critical feature of bacterial cell replication. Cell 172:1271–1293. doi:10.1016/j.cell.2018.01.014 29522747 PMC5870143

[51] Scheu K, Gill R, Saberi S, Meyer P, Emberly E. 2014. Localization of aggregating proteins in bacteria depends on the rate of addition. Front Microbiol 5:418. doi:10.3389/fmicb.2014.00418 25147551 PMC4123723

[52] Meis JF, Chowdhary A. 2018. Candida auris: a global fungal public health threat. Lancet Infect Dis 18:1298–1299. doi:10.1016/S1473-3099(18)30609-1 30293876

[53] Fisher MC, Denning DW. 2023. The WHO fungal priority pathogens list as a game-changer. Nat Rev Microbiol 21:211–212. doi:10.1038/s41579-023-00861-x 36747091 PMC9901396

[54] Rietsch A, Vallet-Gely I, Dove SL, Mekalanos JJ. 2005. ExsE, a secreted regulator of type III secretion genes in Pseudomonas aeruginosa. Proc Natl Acad Sci USA 102:8006–8011. doi:10.1073/pnas.0503005102 15911752 PMC1142391

[55] Sievers F, Wilm A, Dineen D, Gibson TJ, Karplus K, Li W, Lopez R, McWilliam H, Remmert M, Söding J, Thompson JD, Higgins DG. 2011. Fast, scalable generation of high-quality protein multiple sequence alignments using Clustal Omega. Mol Syst Biol 7:539. doi:10.1038/msb.2011.75 21988835 PMC3261699

[56] Letunic I, Bork P. 2016. Interactive tree of life (iTOL) v3: an online tool for the display and annotation of phylogenetic and other trees. Nucleic Acids Res 44:W242–W245. doi:10.1093/nar/gkw290 27095192 PMC4987883

