# Supplementary material for: TssW-PpkA-Fha axis controls the positioning and initiation of the type VI secretion system in Acidovorax citrulli
Source: mBio. 2025 Sep 22;16(11):e01879-25. doi: 10.1128/mbio.01879-25 (PMC12607579; doi:10.1128/mbio.01879-25)
Supplement: Supplemental Material — Supplemental figures, table, and Data S1 caption. [file mbio.01879-25-s0002.pdf]

Supplementary Information

TssW-PpkA-Fha axis controls the positioning and initiation of the type VI secretion system in *Acidovorax citrulli*

Running title: Membrane accessory dictates the whereabouts of T6SS

Tong-Tong Pei[1], Xing-Yu Wang[1, 2], Zi-Yan Ye[1], Yi-Qiu Zhang[1], Jing-Tong Su[1], Han Luo[2], Ya-Jie Zhao[1, 2], Hao-Yu Zheng[1, 2], Zhu Si[3], Ying An[1], Xiaoye Liang[1], Tao Dong[1] *

Affiliations:

[1] School of Life Sciences, Southern University of Science and Technology, Shenzhen, Guangdong, 518055, China

[2] State Key Laboratory of Microbial Metabolism, Joint International Research Laboratory of Metabolic & Developmental Sciences, School of Life Sciences and Biotechnology, Shanghai Jiao Tong University, Shanghai, 200240, China

[3] Cryo-electron Microscopy Center, Southern University of Science and Technology, Shenzhen, Guangdong 518055, China

* Corresponding Email: dongt@sustech.edu.cn

Keywords: protein secretion, LLPS, PpkA, phosphorylation, *Acidovorax*

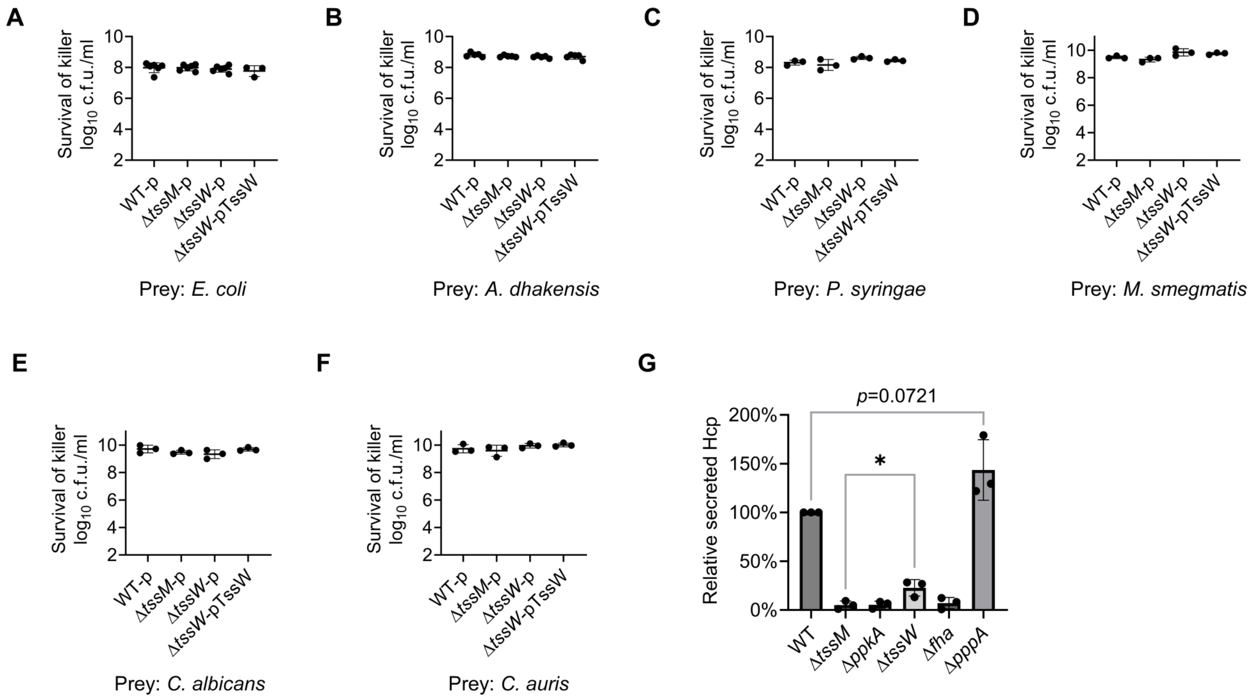

Supplementary Figure 1. Deleting *tssW* leads to an impaired but not abolished T6SS activity. A-F, Survival of killer strains during competition assays for which the survival of the prey is depicted in Figure 1 A-F. **G**, Quantification of secreted Hcp proteins in Figure 1I. Signal intensity of the bands in the Western blotting was quantified using Fiji. Hcp secretion from the wild-type strain was set as 100%, and the signal intensity of the other samples was normalized to the wild-type Hcp secretion level. This analysis includes the data from the samples shown in Figure 1I as well as two additional parallel experiments. Error bars indicate the mean +/- standard deviation of three biological replicates, and statistical significance was calculated using One-way ANOVA test for each group, *$P < 0.05$.

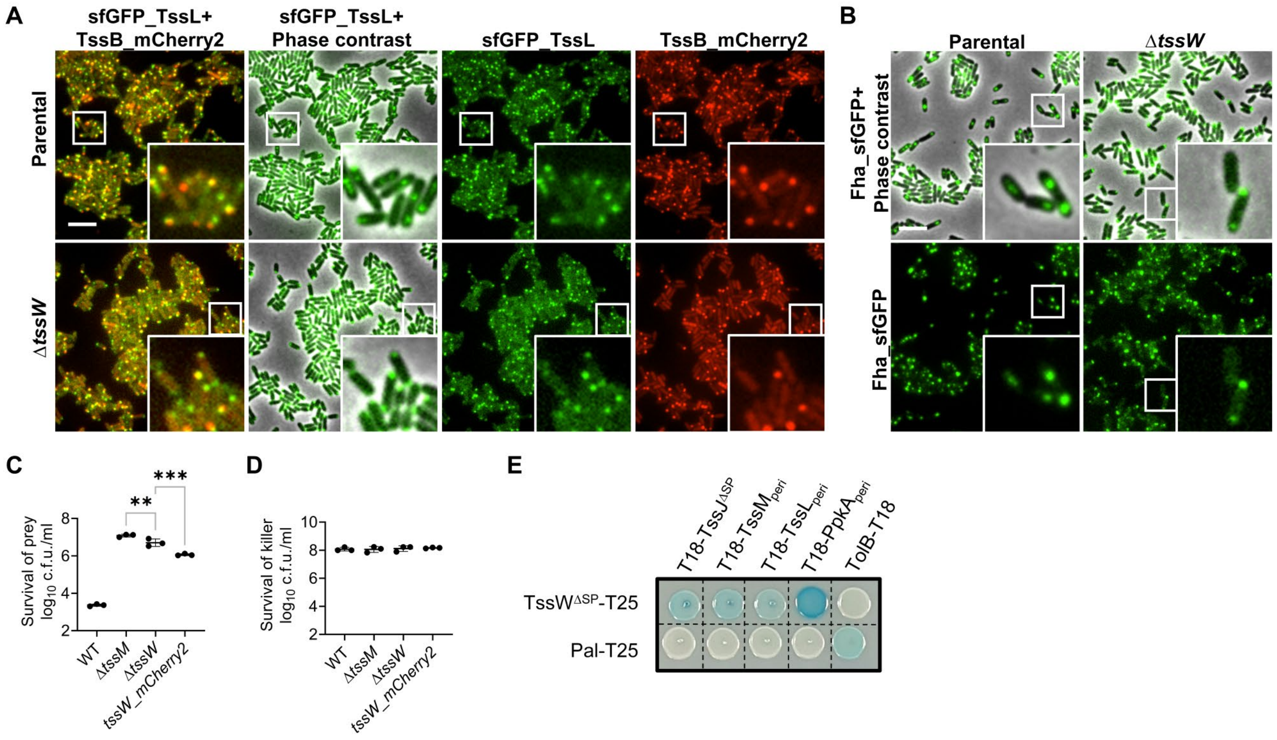

Supplementary Figure 2. Deleting *tssW* results in polar-localized T6SS apparatuses. A, Fluorescence microscopy images showing sfGFP_TssL and TssB_mCherry2 localization in AAC00-1 Parental and Δ*tssW*. **B**, Fluorescence microscopy images showing Fha_sfGFP localization in AAC00-1 Parental and Δ*tssW*. **C**, Competition analysis of the AAC00-1 wild type (WT), T6SS-null mutant Δ*tssM*, Δ*tssW*, and *tssW_mCherry2*. Killer strains are indicated and the prey strain is the *E. coli* MG1655 carrying pPSV37-sfGFP plasmid. Survival of killer strains during competition assays is depicted in **D**. For **C** and **D**, error bars indicate the mean +/- standard deviation of three biological replicates and statistical significance was calculated using One-way ANOVA test for each group, $**P < 0.01$, $***P < 0.001$. **E**, Bacterial two-hybrid analysis of TssW interactions with T6SS membrane components. Proteins fused with the adenylate cyclase T25 or T18 subunits were co-expressed in the BTH101 reporter strain as indicated. A positive interaction is indicated by color development on X-Gal plates. Pal and TolB were used as positive controls.

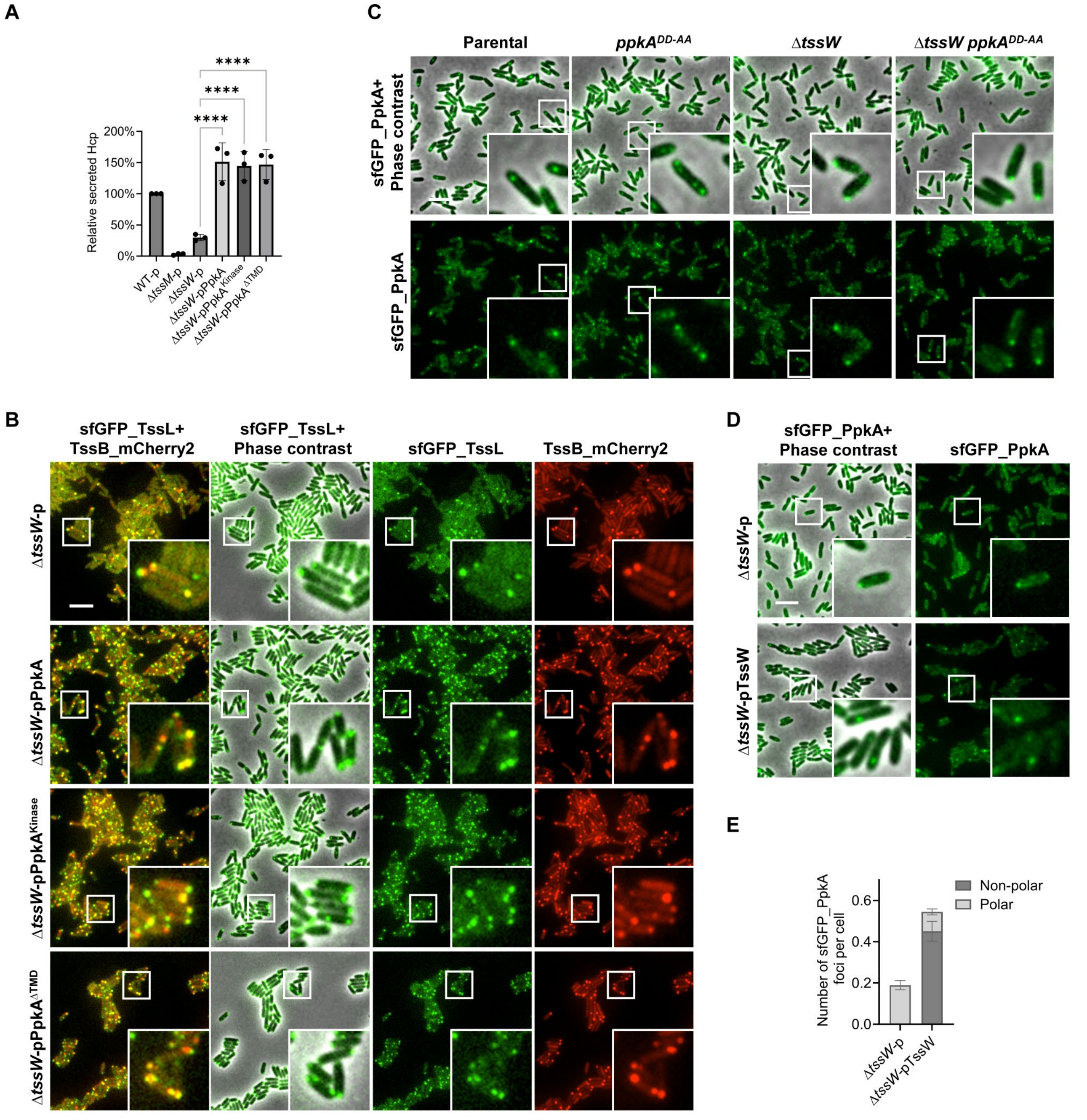

Supplementary Figure 3. Plasmid-borne PpkA and its variants enhance T6SS activity in AAC00-1 Δ*tssW* mutants. A, Quantification of secreted Hcp proteins in Figure 3E. Signal intensity of the bands in the Western blotting was quantified using Fiji. Hcp secretion from the wild-type strain was set as 100%, and the signal intensity of the other samples was normalized to the wild-type Hcp secretion level. This analysis includes the data from the samples shown in Figure 3E as well as two additional parallel experiments. **B**, Fluorescence microscopy images showing sfGFP_TssL and TssB_mCherry2 localization in AAC00-1 Δ*tssW* expressing PpkA, $PpkA^{Kinase}$, or $PpkA^{\Delta TMD}$. **C**, Fluorescence microscopy images showing sfGFP_PpkA localization in AAC00-1 Parental, Δ*tssW*, $ppkA^{DD\text{-}AA}$, and Δ*tssW* $ppkA^{DD\text{-}AA}$. **D**, Fluorescence microscopy images showing sfGFP_PpkA localization in AAC00-1 Δ*tssW* with or without plasmid-borne TssW. For **B-D**, a representative 33- × 33-µm field of cells with

a 3× magnified 5.5- × 5.5-μm inset (marked by box) is shown. Scale bar: 5 μm. **E**, Quantification of cells forming sfGFP_PpkA foci in AAC00-1 Δ*tssW* with or without plasmid-borne TssW. The ratios of polar and non-polar foci are shown in light gray and dark gray, respectively. For **A** and **E**, error bars indicate the mean +/- standard deviation of three biological replicates, and statistical significance was calculated using One-way ANOVA test for each group, ****$P < 0.0001$.

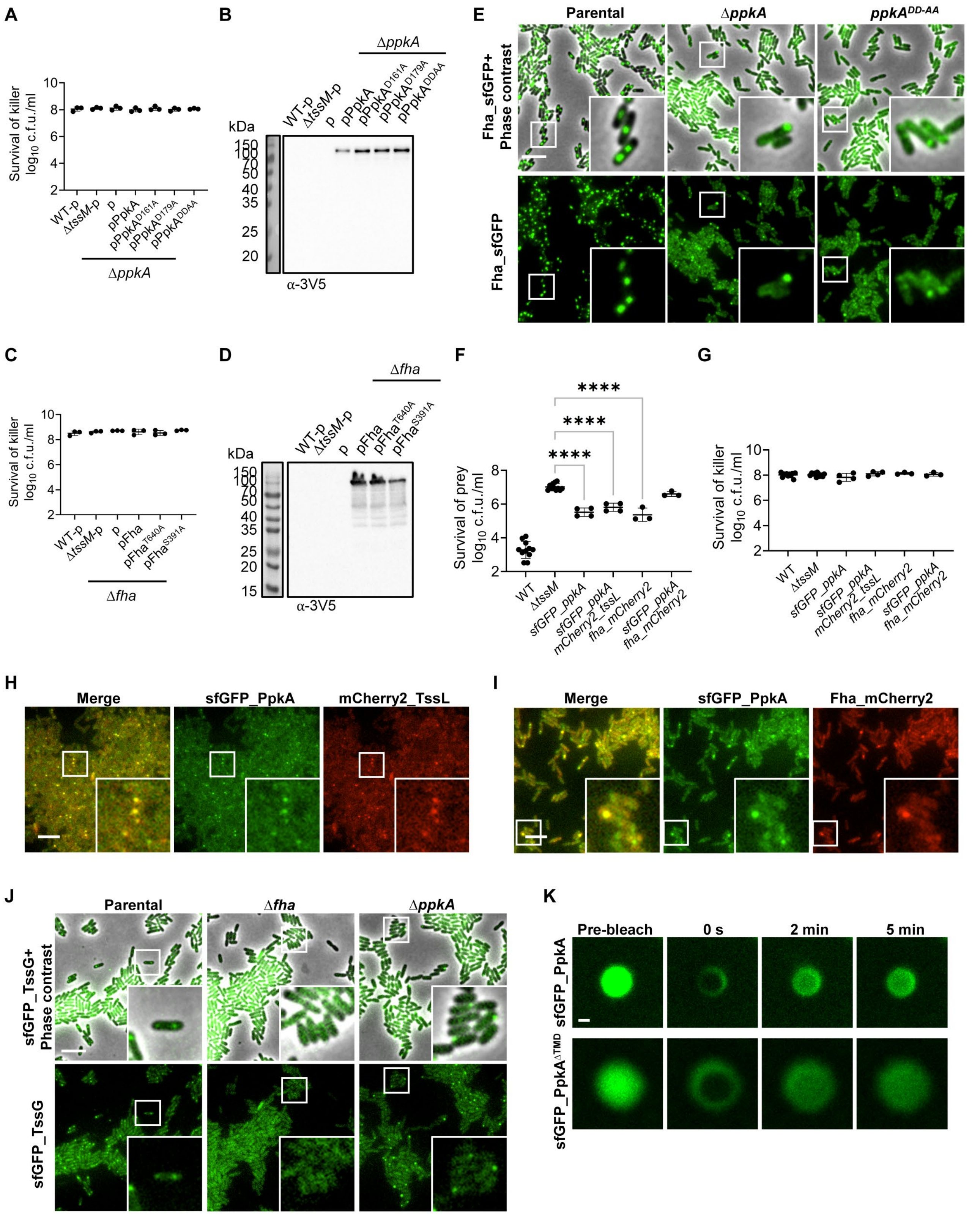

Supplementary Figure 4. PpkA is important for a fully functional T6SS. A, Survival of killer strains during competition assays for which the survival of the prey is depicted in Figure 4B. **B**, Western blotting analysis confirming that the PpkA and its variants used in Figure 4B were expressed. **C**,

Survival of killer strains during competition assays for which the survival of the prey is depicted in Figure 4C. **D**, Western blotting analysis confirming that the Fha and its variants used in Figure 4C were expressed. **E**, Fluorescence microscopy images showing Fha_sfGFP localization in AAC00-1 Parental, Δ*ppkA*, and *ppkA*$^{DD\text{-}AA}$. **F**, Competition analysis of the fluorescence-labeled AAC00-1 strains. Killer strains are indicated and the prey strain is the *E. coli* MG1655 carrying pPSV37-sfGFP plasmid. The wild type (WT) and T6SS-null mutant Δ*tssM* serve as positive and negative control, respectively. Survival of killer strains during competition assays is depicted in **G**. For **A**, **C**, **F**, and **G**, Error bars indicate the mean +/- standard deviation of at least three biological replicates and statistical significance was calculated using One-way ANOVA test for each group, ****P < 0.0001. **H**, Fluorescence microscopy images showing co-localization between sfGFP_PpkA and mCherry2_TssL in AAC00-1. **I**, Fluorescence microscopy images showing co-localization between sfGFP_PpkA and Fha_mCherry2 in AAC00-1. **J**, Fluorescence microscopy images showing sfGFP_TssG localization in AAC00-1 Parental, Δ*fha*, and Δ*ppkA*. For **E**, **H**, **I**, and **J**, a representative 33- × 33-µm field of cells with a 3× magnified 5.5- × 5.5-µm inset (marked by box) is shown. Scale bar: 5 µm. **K**, Representative fluorescence recovery of a photobleached droplet of sfGFP_PpkA or sfGFP_PpkA$^{\Delta TMD}$. A representative 8- × 8-µm field is shown. Scale bar: 1 µm.

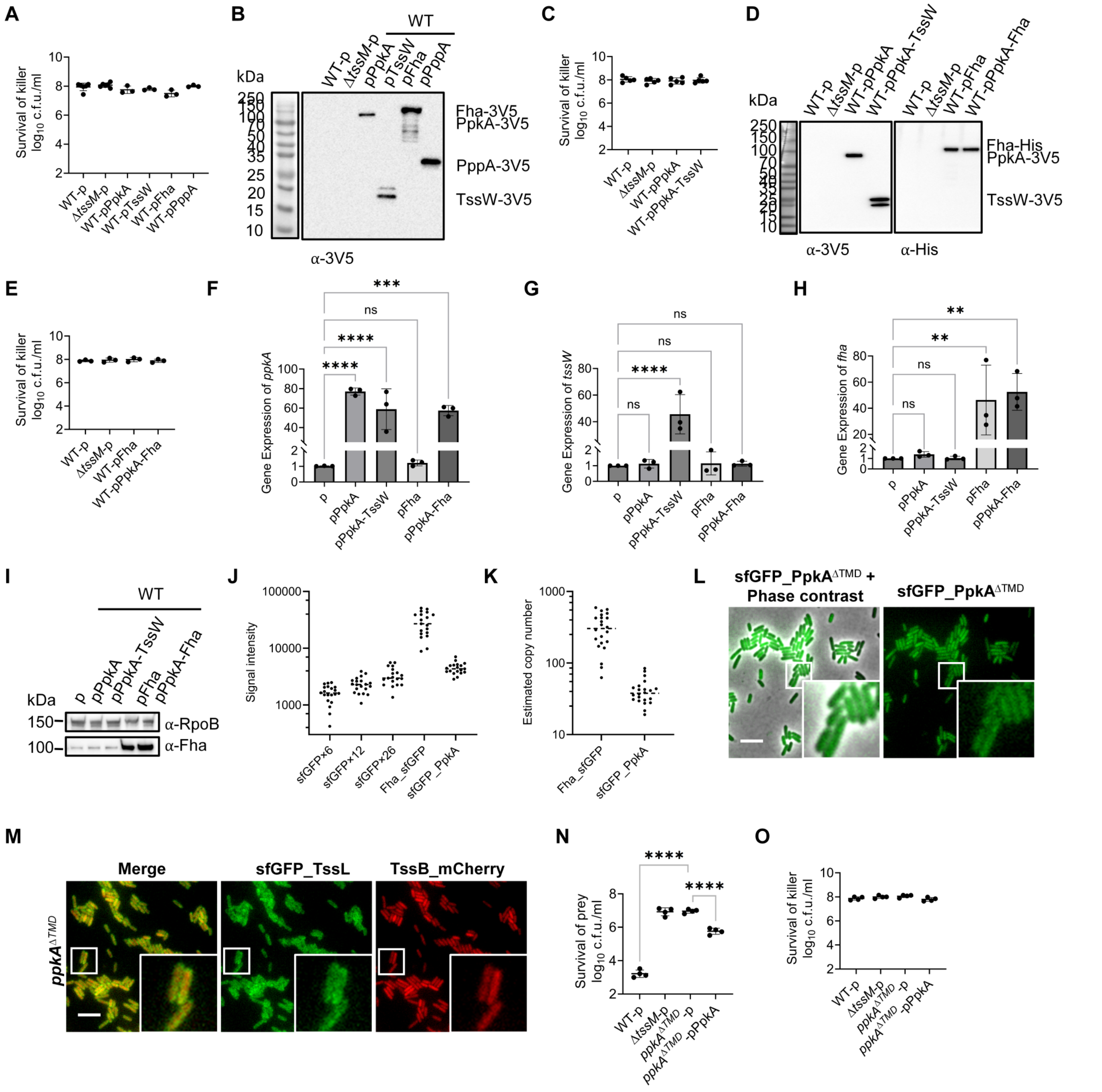

Supplementary Figure 5. Expressing TssW, PpkA, or Fha in AAC00-1 modulates its T6SS assembly. **A**, **C**, and **E**, Survival of killer strains during competition assays for which the survival of the prey is depicted in Figure 6 A, E and F, respectively. **B** and **D**, Western blotting analysis confirming the expression of plasmid-borne PpkA, TssW, Fha, and PppA used in Figure 6. qRT-PCR analysis confirming the transcription of plasmid-borne PpkA (**F**), TssW (**G**), and Fha (**H**) used in Figure 6 B-F. **I**, Western blotting analysis confirming the protein levels of Fha used in Figure 6 B-F. **J**, Quantification of sfGFP fluorescence signals. The distributions of signal intensities measured from raw data for each strain are shown in the graph. The signal distributions of 6×, 12×, and 26× sfGFP correspond to *V. cholerae* V52 strains harboring 3×*lacO*, 6×*lacO*, and 13×*lacO* arrays expressing plasmid-borne LacI^{mut}_sfGFP, respectively. The distributions of signal intensities for Fha_sfGFP and sfGFP_PpkA correspond to the AAC00-1 strain harboring chromosomal fusions of Fha_sfGFP or sfGFP_PpkA. A

standard curve was generated using the average signal intensities of 6×, 12×, and 26× sfGFP to estimate the sfGFP copy numbers in Fha_sfGFP and sfGFP_PpkA, as shown in **K**. **L**, Fluorescence microscopy images showing sfGFP_PpkA $^{\Delta TMD}$ localization in AAC00-1 *sfGFP_ppkA*$^{\Delta TMD}$. **M**, Fluorescence microscopy images showing sfGFP_TssL and TssB_mCherry2 localization in AAC00-1 *ppkA*$^{\Delta TMD}$. For **L** and **M**, a representative 33- × 33-µm field of cells with a 3× magnified 5.5- × 5.5-µm inset (marked by box) is shown. Scale bar: 5 µm. **N**, Competition analysis of the *ppkA*$^{\Delta TMD}$ mutant complemented with plasmid-borne PpkA. Survival of killer strains during competition assays is depicted in **O**. Killer strains are indicated and the prey strain is the *E. coli* MG1655 carrying pPSV37-sfGFP plasmid. The wild type (WT) and T6SS-null mutant Δ*tssM* serve as positive and negative control, respectively. For **A**, **C**, **E-H**, **N**, and **O**, error bars indicate the mean +/- standard deviation of at least three biological replicates and statistical significance was calculated using One-way ANOVA test for each group, $^{**}P < 0.01$, $^{***}P < 0.001$, $^{****}P < 0.0001$, ns, not significant.

Supplementary Table 1. Plasmids, strains, and primers

Plasmid	Description	Reference
pEXG2.0	Suicidal conjugation vector for chromosomal allelic changes in AAC00-1	(1)
pEXG2.0-tssW	Suicidal vector to construct the AAC00-1 in-frame deletion mutant of *tssW*	This study
pEXG2.0-ppkA	Suicidal vector to construct the AAC00-1 in-frame deletion mutant of *ppkA*	This study
pEXG2.0-fha	Suicidal vector to construct the AAC00-1 in-frame deletion mutant of *fha*	(2)
pEXG2.0-pppA	Suicidal vector to construct the AAC00-1 in-frame deletion mutant of *pppA*	This study
pEXG2.0-TssW-mCherry2	Suicidal vector to construct the AAC00-1 mutant with chromosomal insertion TssW_mCherry2	This study
pEXG2.0-sfGFP-PpkA	Suicidal vector to construct the AAC00-1 mutant with chromosomal insertion sfGFP_PpkA	This study
pEXG2.0-PpkA-DD-AA	Suicidal vector to construct the AAC00-1 mutant of $ppkA^{DD\text{-}AA}$	This study
pEXG2.0-tssJ-clpV	Suicidal vector to construct the AAC00-1 in-frame deletion mutant of *tssJ-clpV*	This study
pBAD24	Arabinose inducible expression vector, kanamycin resistance	Lab stock
pBAD24-PhoA	Arabinose inducible expression of PhoA	This study
pBAD24-$PhoA^{\Delta SP}$	Arabinose inducible expression of $PhoA^{\Delta SP}$	This study
pBAD24-TssW-$PhoA^{\Delta SP}$	Arabinose inducible expression of TssW-$PhoA^{\Delta SP}$	This study
pBAD24-$TssW^{\Delta SP}$-$PhoA^{\Delta SP}$	Arabinose inducible expression of TssW-$PhoA^{\Delta SP}$	This study
pBAD24-PpkA-$PhoA^{\Delta SP}$	Arabinose inducible expression of PpkA-$PhoA^{\Delta SP}$	This study
pBAD24-$PhoA^{\Delta SP}$-PpkA	Arabinose inducible expression of $PhoA^{\Delta SP}$-PpkA	This study
pMV261-sfGFP	Temperature Inducible *E. coli-Mycobacterium* shuttle plasmid of pMV261 with sfGFP fusion, kanamycin resistance	Lab stock
pBBR1MCS2	A broad-host-range cloning vector, kanamycin resistance	Lab stock
pBBR1MCS5	A broad-host-range cloning vector, gentamicin resistance	Lab stock
pBBR1MCS2-PpkA-3V5	Constitutive expression of PpkA with a C-terminal 3V5 tag	This study
pBBR1MCS2-$PpkA^{D161A}$-3V5	Constitutive expression of $PpkA^{D161A}$ with a C-terminal 3V5 tag	This study
pBBR1MCS2-$PpkA^{D179A}$-3V5	Constitutive expression of $PpkA^{D179A}$ with a C-terminal 3V5 tag	This study
pBBR1MCS2-$PpkA^{DD\text{-}AA}$-3V5	Constitutive expression of $PpkA^{DD\text{-}AA}$ with a C-terminal 3V5 tag	This study
pBBR1MCS2-$PpkA^{Kinase}$-3V5	Constitutive expression of $PpkA^{Kinase}$ with a C-terminal 3V5 tag	This study
pBBR1MCS2-$PpkA^{\Delta TMD}$ -3V5	Constitutive expression of $PpkA^{\Delta TMD}$ with a C-terminal 3V5 tag	This study
pBBR1MCS2-Fha-3V5	Constitutive expression of Fha with a C-terminal 3V5 tag	(2)
pBBR1MCS2-Fha^{T640A}-3V5	Constitutive expression of Fha^{T640A} with a C-terminal 3V5 tag	This study
pBBR1MCS2-Fha^{S391A}-3V5	Constitutive expression of Fha^{S391A} with a C-terminal 3V5 tag	This study
pBBR1MCS2-SP^{AcrB}-sfGFP	Constitutive expression of SP^{AcrB}-sfGFP	This study

pBBR1MCS2-TssW-FLAG	Constitutive expression of TssW with a C-terminal FLAG tag	This study
pBBR1MCS2-PppA-3V5	Constitutive expression of PppA with a C-terminal 3V5 tag	This study
pBBR1MCS2-PpkA-TssW-3V5	Constitutive expression of PpkA and TssW with a C-terminal 3V5 tag	This study
pBBR1MCS2-Fha-His	Constitutive expression of Fha with a C-terminal His tag	This study
pBBR1MCS2-PpkA-Fha-His	Constitutive expression of PpkA and Fha with a C-terminal His tag	This study
pBAD24Kan-MBP-FLAG	Arabinose inducible expression of MBP with a C-terminal FLAG tag	Lab stock
pETSUMO	IPTG inducible expression of His-SUMO	Lab stock
pETSUMO-TssM	IPTG inducible expression of TssM with an N-terminal His-SUMO tag	(2)
pETSUMO-TssE	IPTG inducible expression of TssE with an N-terminal His-SUMO tag	(2)
pETSUMO-TssF	IPTG inducible expression of TssF with an N-terminal His-SUMO tag	(2)
pETSUMO-TssG	IPTG inducible expression of TssG with an N-terminal His-SUMO tag	(2)
pETSUMO-TssK	IPTG inducible expression of TssK with an N-terminal His-SUMO tag	(2)
pET22b-Fha-sfGFP-2strep	IPTG inducible expression of Fha_sfGFP with a C-terminal 2strep tag	(2)
pET22b-TssJ -His	IPTG inducible expression of TssJ with a C-terminal His tag	(2)
pET22b-TssL-His	IPTG inducible expression of TssL with a C-terminal His tag	(2)
pET22b-TssA-His	IPTG inducible expression of TssA with a C-terminal His tag	(2)
pET22b-PpkA-His	IPTG inducible expression of PpkA with a C-terminal His tag	This study
pET22b-PpkA-FLAG	IPTG inducible expression of PpkA with a C-terminal FLAG tag	This study
pET22b-PpkA$^{DD\text{-}AA}$-His	IPTG inducible expression of PpkA$^{DD\text{-}AA}$ with a C-terminal His tag	This study
pET22b-PpkA$^{DD\text{-}AA}$-FLAG	IPTG inducible expression of PpkA$^{DD\text{-}AA}$ with a C-terminal FLAG tag	This study
pETAmp-2strep-sfGFP-PpkA	IPTG inducible expression of sfGFP_PpkA with a N-terminal 2strep tag	This study
pETAmp-2strep-sfGFP-PpkA$^{\Delta TMD}$	IPTG inducible expression of sfGFP-PpkA$^{\Delta TMD}$ with a N-terminal 2strep tag	This study
pETAmp-2strep-PpkA$^{\Delta TMD}$	IPTG inducible expression of PpkA$^{\Delta TMD}$ with a N-terminal 2strep tag	This study
pETAmp-2strep-sfGFP	IPTG inducible expression of sfGFP with a N-terminal 2strep tag	(2)
pETAmp-2strep-sfGFP-MBP	IPTG inducible expression of sfGFP-MBP with a N-terminal 2strep tag	(2)
pETAmp-2strep-TssW$^{\Delta SP}$-sfGFP	IPTG inducible expression of TssW$^{\Delta SP}$-sfGFP with a N-terminal 2strep tag	This study
pETAmp-2strep-TssJ$^{\Delta SP}$-sfGFP	IPTG inducible expression of TssJ$^{\Delta SP}$-sfGFP with a N-terminal 2strep tag	This study
pETAmp-2strep-sfGFP-TssL$_{cyto}$	IPTG inducible expression of sfGFP-TssL$_{cyto}$ with a N-terminal 2strep tag	(2)

**Strain**	**Genotype**	**Description**	**Reference**
Acidovorax citrulli AAC00-1	AAC00-1	Reference strain of Group II strain	(3)
	Δ*tssM*	In-frame deletion of *tssM*	(3)

	Δ*fha*	In-frame deletion of *fha*	(2)
	Δ*tssW*	In-frame deletion of *tssW*	This study
	Δ*ppkA*	In-frame deletion of *ppkA*	This study
	Δ*pppA*	In-frame deletion of *pppA*	This study
	sfGFP_tssL tssB_mCherry2	Chromosomal fusion of the sfGFP_TssL and TssB_mCherry2	(2)
	sfGFP_tssL tssB_mCherry2 Δ*tssW*	*sfGFP_tssL tssB_mCherry2* with in-frame deletion of *tssW*	This study
	sfGFP_tssL tssB_mCherry2 Δ*ppkA*	*sfGFP_tssL tssB_mCherry2* with in-frame deletion of *ppkA*	This study
	sfGFP_tssL tssB_mCherry2 $ppkA^{DD\text{-}AA}$	*sfGFP_tssL tssB_mCherry2* with chromosomal mutation of $\mathrm{PpkA}^{DD\text{-}AA}$	This study
	tssW_mCherry2	Chromosomal fusion of the TssW_mCherry2	This study
	fha_sfGFP	Chromosomal fusion of the Fha_sfGFP	(2)
	Δ*ppkA fha_sfGFP*	*fha_sfGFP* with in-frame deletion of *ppkA*	This study
	$ppkA^{DD\text{-}AA}$ *fha_sfGFP*	*fha_sfGFP* with chromosomal mutation of $\mathrm{PpkA}^{DD\text{-}AA}$	This study
	fha^{T640A}*_sfGFP*	*fha_sfGFP* with chromosomal mutation of Fha^{T640A}	This study
	Δ*tssJ fha_sfGFP*	*fha_sfGFP* with in-frame deletion of *tssJ*	This study
	Δ*tssL fha_sfGFP*	*fha_sfGFP* with in-frame deletion of *tssL*	This study
	Δ*tssG fha_sfGFP*	*fha_sfGFP* with in-frame deletion of *tssG*	This study
	Δ*tssA fha_sfGFP*	*fha_sfGFP* with in-frame deletion of *tssA*	This study
	Δ*tssB fha_sfGFP*	*fha_sfGFP* with in-frame deletion of *tssB*	This study
	Δ*hcp fha_sfGFP*	*fha_sfGFP* with in-frame deletion of *hcp*	This study
	Δ*tssJ-clpV fha_sfGFP*	*fha_sfGFP* with in-frame deletion of *tssJ-clpV*	This study
	Δ*tssW fha_sfGFP*	*fha_sfGFP* with in-frame deletion of *tssW*	This study
	sfGFP_ppkA	Chromosomal fusion of the sfGFP_PpkA	This study
	sfGFP_ppkA mCherry2_tssL	Chromosomal fusion of the sfGFP_PpkA and mCherry2_TssL	This study
	sfGFP_ppkA fha_mCherry2	Chromosomal fusion of the sfGFP_PpkA and Fha_mCherry2	This study
	*sfGFP_*$ppkA^{DD\text{-}AA}$	*sfGFP_ppkA* with chromosomal mutation of $\mathrm{PpkA}^{DD\text{-}AA}$	This study
	Δ*fha sfGFP_ppkA*	*sfGFP_ppkA* with in-frame deletion of *fha*	This study
	Δ*tssJ sfGFP_ppkA*	*sfGFP_ppkA* with in-frame deletion of *tssJ*	This study
	Δ*tssM sfGFP_ppkA*	*sfGFP_ppkA* with in-frame deletion of *tssM*	This study
	Δ*tssL sfGFP_ppkA*	*sfGFP_ppkA* with in-frame deletion of *tssL*	This study
	Δ*tssF sfGFP_ppkA*	*sfGFP_ppkA* with in-frame deletion of *tssF*	This study
	Δ*tssB sfGFP_ppkA*	*sfGFP_ppkA* with in-frame deletion of *tssB*	This study
	Δ*hcp sfGFP_ppkA*	*sfGFP_ppkA* with in-frame deletion of *hcp*	This study

	Δ*tssJ-clpV sfGFP_ppkA*	*sfGFP_ppkA* with in-frame deletion of *tssJ-clpV*	This study
	Δ*tssW sfGFP_ppkA*	*sfGFP_ppkA* with in-frame deletion of *tssW*	This study
	sfGFP_tssG	Chromosomal fusion of the sfGFP_TssG	(2)
	Δ*fha sfGFP_tssG*	*sfGFP_tssG* with in-frame deletion of *fha*	(2)
	Δ*ppkA sfGFP_tssG*	*sfGFP_tssG* with in-frame deletion of *ppkA*	This study
Escherichia coli			
T-Fast		Strain used for cloning and gene expression	TIANGEN
WM6026		Strain used for conjugation	Lab stock
BL21(DE3)		Strain used for protein expression	Lab stock
MG1655		Strain used for competition assay	Lab stock
Aeromonas dhakensis SSU		Strain used for competition assay	Lab stock
Pseudomonas syringae pv. *syringae*		Strain used for competition assay	Lab stock
Mycobacterium smegmatis mc^{2}155		Strain used for competition assay	Lab stock
Candida albicans		Strain used for competition assay	Lab stock
Candida auris		Strain used for competition assay	Lab stock

**Primer**	**Sequence (5'-3')**	**Description**
pEXG2.0-hifi-f	agatctcagagtcgacctgcagaa	Forward primer to amplify pEXG2.0 vector
pEXG2.0-hifi-r	gctcgagctcgaattcggta	Reverse primer to amplify pEXG2.0 vector
pEXG2.0-f	ctgttgcatgggcataaagttgc	Forward confirmation primer of pEXG2.0 vector
pEXG2.0-r	cttcacgttcgctcgcgtat	Reverse confirmation primer of pEXG2.0 vector
PpkA-KO1	taccgaattcgagctcgagcccgggggaatgcgtaattcaccaggac	Forward primer to amplify the upstream of *Aave_1466* for constructing in-frame deletion of *Aave_1466*
PpkA-KO2	tgggtcagcaccacggattcaggcagttcgcggagcca	Reverse primer to amplify the upstream of *Aave_1466* for constructing in-frame deletion of *Aave_1466*
PpkA-KO3	agtggctccgcgaactgcctgaatccgtggtgctgaccc	Forward primer to amplify the downstream of *Aave_1466* for constructing in-frame deletion of *Aave_1466*
PpkA-KO4	ctgcaggtcgactctgagatctcggaatgcaggacgtaggg	Reverse primer to amplify the downstream of *Aave_1466* for constructing in-frame deletion of *Aave_1466*
PpkA-KO5	gcgaagcgaagaatcaggc	Forward primer to confirm the in-frame deletion of *Aave_1466*

PpkA-KO6	atggctacgggaacggaaac	Reverse primer to confirm the in-frame deletion of *Aave_1466*
TssW-KO1	taccgaattcgagctcgagcccgttccggtgccttcg	Forward primer to amplify the upstream of *Aave_1467* for constructing in-frame deletion of *Aave_1467*
TssW-KO2	cgtcttagatgccatgcttacctc	Reverse primer to amplify the upstream of *Aave_1467* for constructing in-frame deletion of *Aave_1467*
TssW-KO3	aagaaggcgccctgaccc	Forward primer to amplify the downstream of *Aave_1467* for constructing in-frame deletion of *Aave_1467*
TssW-KO4	ctgcaggtcgactctgagatctgtccaccgcgaaaagatcg	Reverse primer to amplify the downstream of *Aave_1467* for constructing in-frame deletion of *Aave_1467*
TssW-KO5	aggacatcccgccttcgag	Forward primer to confirm the in-frame deletion of *Aave_1467*
TssW-KO6	ggcatcctttttctcctcgc	Reverse primer to confirm the in-frame deletion of *Aave_1467*
PppA-KO1	taccgaattcgagctcgagcccttgacgtccttgccgat	Forward primer to amplify the upstream of *Aave_1469* for constructing in-frame deletion of *Aave_1469*
PppA-KO2	tcgaccttggtgccttcgg	Reverse primer to amplify the upstream of *Aave_1469* for constructing in-frame deletion of *Aave_1469*
PppA-KO3	gatatggaagatcacgtcctggc	Forward primer to amplify the downstream of *Aave_1469* for constructing in-frame deletion of *Aave_1469*
PppA-KO4	ctgcaggtcgactctgagatctgaggatggcgcctgacag	Reverse primer to amplify the downstream of *Aave_1469* for constructing in-frame deletion of *Aave_1469*
PppA-KO5	tcatggtgcggaaagctgaac	Forward primer to confirm the in-frame deletion of *Aave_1469*
PppA-KO6	cagcgtgtggaaggactcg	Reverse primer to confirm the in-frame deletion of *Aave_1469*
sfGFP-X-hifif	tctaaaggtgaagaactgttcaccg	Forward primer to amplify *sfGFP*
sfGFP-X-hifir	ctgcggccgccttgtacagctcgtccatgc	Reverse primer to amplify *sfGFP*
sfGFP-PpkA-KI1	taccgaattcgagctcgagccgagctttctgcacccttgc	Forward primer to amplify the upstream of *Aave_1466* for constructing chromosomal *sfGFP_ppkA*
sfGFP-PpkA-KI2	cttcacctttagacatgagttcacgcttccaagcacc	Reverse primer to amplify the upstream of *Aave_1466* for constructing chromosomal *sfGFP_ppkA*
sfGFP-PpkA-KI3	gtacaaggcggccgcaggaggaggaatggacaaggaattcagtggctc	Forward primer to amplify *Aave_1466* for constructing chromosomal *sfGFP_ppkA*
sfGFP-PpkA-KI4	ctgcaggtcgactctgagatctccaaatgcgaagggatcatg	Reverse primer to amplify *Aave_1466* for constructing chromosomal *sfGFP_ppkA*
sfGFP-PpkA-KI5	gcgaatacgccgaacactg	Forward primer to confirm the chromosomal *sfGFP_ppkA*
sfGFP-PpkA-KI6	gttgcaggaaggcgtcatc	Reverse primer to confirm the chromosomal *sfGFP_ppkA*
TssW-mCherry2-KI1	taccgaattcgagctcgagcagggtgatcctgcgtgtcatg	Forward primer to amplify the upstream of *Aave_1467* and *Aave_1467* for constructing chromosomal *TssW_mCherry2*
TssW-mCherry2-KI2	ggccgcaggagccttcttggccgggag	Reverse primer to amplify the upstream of *Aave_1467* and *Aave_1467* for constructing chromosomal *TssW_mCherry2*
TssW-mCherry2-KI3	tgaccccggcgggtcgtagcggacatggac	Forward primer to amplify the downstream of *Aave_1467* for constructing chromosomal *TssW_mCherry2*

TssW-mCherry2-KI4	ctgcaggtcgactctgagatctgtccaccgcgaaaagatcg	Reverse primer to amplify the downstream of *Aave_1467* for constructing chromosomal *TssW_mCherry2*
TssW-mCherry2-KI5	gatgccgcagcctctgcac	Forward primer to confirm the chromosomal *TssW_mCherry2*
TssW-mCherry2-KI6	ggcgatctcgctcaggctctta	Reverse primer to confirm the chromosomal *TssW_mCherry2*
TssW-mCherry2-hifif	caagaaggcTccTgcggccgcaggaggagga	Forward primer to amplify *mCherry2*
TssW-mCherry2-hifir	tacgacccgccggggtcacttgtacagctcgtccatgcc	Reverse primer to amplify *mCherry2*
pBBR-PpkA-3V5-F	ggattaggaataggtttaccctcgaatttgtgggtcagcac	Forward primer to amplify *Aave_1466*
pBBR-PpkA-3V5-R	atttcacacaggaaacagctatggacaaggaattcagtggctc	Reverse primer to amplify *Aave_1466*
PpkA-D161A-fd	ctatcaccgcgctatagcgccggacaacatcttcct	Forward primer to amplify *Aave_1466*
PpkA-D161A-rv	gcgctatagcgcggtgatagcagtctgcc	Reverse primer to amplify *Aave_1466*
PpkA -D179A-fd	ctgctggcatttggtgctgcacggcgcatcatcggcgatatg	Forward primer to amplify *Aave_1466*
PpkA-D179A-rv	cagcaccaaatgccagcagcaccggcatgcc	Reverse primer to amplify *Aave_1466*
PpkA-ΔTMD -fd	gacctgcgcaggcatccagcgaggc	Forward primer to amplify *Aave_1466*
PpkA-ΔTMD -rv	tggatgcctgcgcaggtcgcccggacttcg	Reverse primer to amplify *Aave_1466*
PpkA-kinase-v5hifi-r	ggattaggaataggtttaccagaggttgcggatgcctcg	Reverse primer to amplify *Aave_1466*
Fha-T640A-fd	cgtgctggtgtcgctgtcatttcctccggcgcgaacaa	Forward primer to amplify *Aave_1468*
Fha-T640A-rv	atgacagcgacaccagcacggaattcgcgcttgatctc	Reverse primer to amplify *Aave_1468*
Fha-S391A-fd	ggtagcgaagctgcagctgcactggtgcccgaacgttc	Forward primer to amplify *Aave_1468*
Fha-S391A-rv	gcagctgcagcttcgctacctcccgtgaagagccgcagc	Reverse primer to amplify *Aave_1468*
PppA-pBBR2-hifi-f	atttcacacaggaaacagctgtgatcttccatatcgcaggcgag	Forward primer to amplify *Aave_1469*
PppA -v5-hifi-r	ggattaggaataggtttaccggcgccatcctcgctgtcc	Reverse primer to amplify *Aave_1469*
TssW-pBBR2-hifi-f	atttcacacaggaaacagctatggcatctaagacgaagctcattgg	Forward primer to amplify *Aave_1467*
TssW-v5-hifi-r	ggattaggaataggtttaccgggcgccttcttggccg	Reverse primer to amplify *Aave_1467*
pBBR2-hifiF	agctgtttcctgtgtgaaattg	Forward primer to amplify pBBRMCS2 vector
pBBR2-3V5-hifiR	ggtaaacctattcctaatcctctcct	Reverse primer to amplify pBBRMCS2 vector
pBBR2-f	agcgcaacgcaattaatgtgag	Forward confirmation primer of pBBRMCS2 vector
pBBR2-r	catcgcagtacggcctattgg	Reverse confirmation primer of pBBRMCS2 vector
pBBR-Fha-3V5-F	ggattaggaataggtttacccgtcctggccttcgccg	Forward primer to amplify *Aave_1468*
pBBR-Fha-3V5-R	atttcacacaggaaacagctatggacaaagtcgaactcagggtcg	Reverse primer to amplify *Aave_1468*
TssW-flag-hifir	tgtcatcgtcgtccttgtaatcagacgagggcgccttcttggccg	Reverse primer to amplify *Aave_1467*
pETDuet-f	cacgatgcgtccggcgtagagg	Forward confirmation primer of pET vector
pETDuet-r	ggttatgctagttattgctcagcggt	Reverse confirmation primer of pET vector
pET22b-hifi-f	aagcttgcggccgcactcga	Forward primer to amplify pET22b vector

pET22b-hifi-r	catatgtatatctccttcttaaagttaaacaaaattatttctagagg	Reverse primer to amplify pET22b vector
22b-PpkA-f	ttaagaaggagatatacatatggacaaggaattcagtggctc	Forward primer to amplify *Aave_1466*
22b-PpkA-r	tcgagtgcggccgcaagcttctcgaatttgtgggtcagcac	Reverse primer to amplify *Aave_1466*
petstrep-hifi-f	tgataacactgagatccggctgctaacaaag	Forward primer to amplify pET vector
petstrep-hifir	accgctgccggatttttcgaactg	Reverse primer to amplify pET vector
sfgfp-strep-hifif	tcgaaaaatccggcagcggttctaaaggtgaagaactgttcaccggtg	Forward primer to amplify *sfGFP*
PpkA-strep-hifif	ccggcagcggtgacaaggaattcagtggctccg	Forward primer to amplify *Aave_1466*
PpkA-strep-hifir	agccggatctcagtgttatcactcgaatttgtgggtcagca	Reverse primer to amplify *Aave_1466*
TssJ-strephifif	tcgaaaaatccggcagcggttgcggcatgatcagcaacct	Forward primer to amplify *Aave_1470*
TssJ-linkerhifir	atcctcctcctgcagcggcaggagcggtctggatcttcacc	Reverse primer to amplify *Aave_1470*
TssW-strephifif	tcgaaaaatccggcagcggttgccagacgccccctgc	Forward primer to amplify *Aave_1467*
TssW-linkerhifir	atcctcctcctgcagcggcgggcgccttcttggccg	Reverse primer to amplify *Aave_1467*

Supplementary Data 1. Sequence file of TssM homologs for Figure 1H.

Note for references

1. A. Rietsch, I. Vallet-Gely, S. L. Dove, J. J. Mekalanos, ExsE, a secreted regulator of type III secretion genes in *Pseudomonas aeruginosa*. *Proc. Natl. Acad. Sci. U. S. A.* **102**, 8006–8011 (2005).
2. T.-T. Pei, *et al.*, Fha initiates the inside-out assembly of the type VI secretion system. *Cell Rep.* **44**, 115990 (2025).
3. T.-T. Pei, *et al.*, Delivery of an Rhs-family nuclease effector reveals direct penetration of the Gram-positive cell envelope by a type VI secretion system in *Acidovorax citrulli*. *mLife* **1**, 66–78 (2022).
